# Visual Impairment and Cardiovascular Risk Factors in Hispanic and Latino Adults

**DOI:** 10.1001/jamanetworkopen.2026.17975

**Published:** 2026-06-12

**Authors:** Charlotte E. Joslin, Angie Wang, David J. Lee, Lawrence J. Ulanski, Byron L. Lam, Thasarat S. Vajaranant, Carlos E. Mendoza-Santiesteban, Michael L. Stewart, Giselle Gutierrez Savoy, Mehmet Cem Mocan, Franklyn Gonzalez, D. Diane Zheng, Bharat Thyagarajan, Laura Coco, Amber Pirzada, Jianwen Cai, Martha L. Daviglus

**Affiliations:** 1 Department of Ophthalmology and Visual Sciences, College of Medicine, University of Illinois Chicago; 2 Division of Epidemiology and Biostatistics, School of Public Health, University of Illinois Chicago; 3 Department of Public Health Sciences, Miller School of Medicine, University of Miami, Miami, Florida; 4 Bascom Palmer Eye Institute, Miller School of Medicine, University of Miami, Miami, Florida; 5 Department of Biostatistics, University of North Carolina at Chapel Hill; 6 Department of Psychiatry and Behavioral Sciences, Miller School of Medicine, University of Miami, Miami, Florida; 7 Department of Laboratory Medicine and Pathology, University of Minnesota, Minneapolis; 8 School of Speech, Language, and Hearing Sciences, San Diego State University, San Diego, California; 9 Institute for Minority Health Research, College of Medicine, University of Illinois Chicago

## Abstract

**Importance:**

Visual impairment (VI) affects approximately 7 million individuals in the US and is associated with adverse outcomes; cardiovascular disease (CVD) risk factors and nonmedical drivers of health (NMDOH) may contribute. Prior research in Mexican American individuals suggested high VI prevalence, but data across more diverse US Hispanic/Latino populations remain limited.

**Objective:**

To estimate VI prevalence and assess its associations with CVD risk factors in Hispanic/Latino adults in the US.

**Design, Setting, and Participants:**

This cross-sectional study analyzed data from SOL Ojos, an ancillary study of the Hispanic Community Health Study/Study of Latinos (HCHS/SOL). Participants were Hispanic/Latino adults aged 40 years or older from community-based cohorts in Chicago, Illinois, and Miami, Florida, who completed HCHS/SOL visit 3 (2020-2024) and were recruited into SOL Ojos from July 1, 2021, to July 1, 2023.

**Exposures:**

Individual and cumulative major CVD risk factors: diabetes, hypercholesterolemia, hypertension, obesity, and smoking.

**Main Outcome and Measures:**

Age-standardized VI (visual acuity less than or equal to 20/40 in the better-seeing eye), assessed by habitual visual acuity, best-corrected visual acuity, and uncorrected refractive error (correctable to more than 20/40). Odds ratios (ORs) for VI by CVD risk factors were estimated using logistic regression, sequentially adjusting for traditional, biologic, and NMDOH-related confounders. Age-standardized weighted estimates reflected the target population.

**Results:**

Among 3288 participants (mean age, 58.7 [95% CI, 57.9-59.5] years; 61.9% [95% CI, 60.2%-63.5%] female), VI prevalence by habitual visual acuity was 7.1% (95% CI, 5.3%-8.8%) in men vs 7.0% (95% CI, 5.2%-8.8%) in women and by best-corrected visual acuity was 1.8% (95% CI, 1.0%-2.6%) in men vs 2.6% (95% CI, 1.6%-3.7%) in women; VI by uncorrected refractive error was 5.2% (95% CI, 3.7%-6.8%) in men vs 4.4% (95% CI, 3.0%-5.8%) in women. VI prevalence varied across Hispanic/Latino backgrounds in men (habitual visual acuity VI range, 4.1% [95% CI, 1.8%-6.4%] to 16.9% [95% CI, 11.6%-22.2%]; uncorrected refractive error range, 2.3% [95% CI, 0.6%-4.0%] to 13.5% [95% CI, 8.1%-18.9%]) and was highest among Mexican men. In fully adjusted models, diabetes was associated with VI assessed by best-corrected visual acuity (OR, 4.65; 95% CI, 2.42-8.91), as were cumulative CVD risk factors vs 1 or none (3 factors: OR, 3.47 [95% CI, 1.45-8.33]; ≥4 factors: OR, 9.80 [95% CI, 4.34-22.13]). ORs were smaller when VI was assessed by habitual visual acuity (eg, diabetes OR, 2.03 [95% CI, 1.37-3.01]).

**Conclusions and Relevance:**

In this cross-sectional study of US Hispanic/Latino adults, VI and CVD risk factor prevalence varied by ethnic background, and diabetes and cumulative CVD risk factors were associated with VI. The findings provide updated prevalence estimates and support the need for longitudinal studies of causal relationships between VI and CVD risk factors.

## Key Points

**Question:**

What is the prevalence of visual impairment (VI) and its association with cardiovascular disease (CVD) risk factors among Hispanic/Latino US adults with diverse backgrounds?

**Findings:**

In this cross-sectional study of 3288 Hispanic/Latino adults aged 40 years or older, age-standardized VI prevalence and CVD burden varied by Hispanic/Latino background. Diabetes and having 3 or more cumulative CVD risk factors were associated with VI assessed by both best-corrected and habitual visual acuity but not uncorrected refractive error.

**Meaning:**

The findings suggest VI is prevalent and associated with cardiometabolic burden in US Hispanic/Latino adults, highlighting opportunities for longitudinal research investigating incident disease and causality.

## Teaser

This cross-sectional study estimates the prevalence of visual impairment in US Hispanic and Latino adults with diverse backgrounds and explores whether it is associated with risk factors for cardiovascular disease.

## Introduction

Visual impairment (VI) affects approximately 7 million people in the US.^1^ Objectively measured VI is associated with adverse health outcomes, including mortality,^2,3,4,5,6,7^ and ranges from mild impairment to blindness. VI results from eye diseases of various etiologies and uncorrected refractive error, which is a leading cause.^8,9,10,11,12,13,14,15^ High VI prevalence is observed in US Hispanic/Latino populations,^8,9,10,11^ with most studies focusing on Mexican populations.^8,9,10,11^ Between 2000 and 2024, the Hispanic/Latino population accounted for 56% of US population growth, reaching 68 million (20%) and becoming the largest racial and ethnic minority population.^16^

The landmark Hispanic Community Health Study/Study of Latinos (HCHS/SOL), a population-based cohort, was designed to examine chronic disease risk factors in diverse Hispanic/Latino populations including Central American, Cuban, Dominican, Mexican, Puerto Rican, South American, and other backgrounds.^17,18^ Baseline findings (2008-2011)^19^ demonstrated variation in the prevalence of major cardiovascular disease (CVD) risk factors (diabetes, hypertension, smoking, obesity, and hypercholesterolemia) across Hispanic or Latino backgrounds, with notable burden in all groups.^20,21,22,23,24,25,26^ Overlapping risk factors for chronic eye disease and CVD suggest there may be variation in VI prevalence across Hispanic/Latino groups.

The Study of Latinos (SOL) Ojos was designed to assess age-standardized prevalence of chronic eye diseases and associated risk factors among HCHS/SOL participants aged 40 years or older. The current study aimed to characterize the VI prevalence (assessed by habitual visual acuity, best corrected visual acuity, and uncorrected refractive error) across Hispanic/Latino adults; examine associations between CVD risk factors and VI; and assess confounding by nonmedical drivers of health (NMDOH)^27^ via sequential modeling in order to provide estimates of VI burden in US Hispanic/Latino adults and clarify whether NMDOH attenuate the association between CVD risk factors and prevalent VI.

## Methods

### Study Sample

This cross-sectional study analyzed data from SOL Ojos, a study supported by the National Institutes of Health to add comprehensive eye data to HCHS/SOL, enabling VI assessment across diverse Hispanic/Latino populations. The HCHS/SOL design and sampling methods were described previously.^17,18^ In brief, HCHS/SOL enrolled 16 415 adults aged 18 to 74 years at visit 1 (2008-2011) recruited from 4 US communities (Bronx, New York; Chicago, Illinois; Miami, Florida; and San Diego, California) using a stratified multistage area-probability design. From July 1, 2021, to July 1, 2023, SOL Ojos recruited all participants aged 40 years or older in Chicago and Miami who completed HCHS/SOL visit 3 (2020-2024), with a target of 3000 individuals. The current study was reviewed and approved by the single institutional review board at the University of North Carolina at Chapel Hill. Written informed consent was obtained. Reporting followed the Strengthening the Reporting of Observational Studies in Epidemiology (STROBE) guideline for cross-sectional studies.^28^

### Examination Methods and Outcome and VI Definitions

SOL Ojos participants underwent comprehensive eye examinations, with data recorded in the Research Electronic Data Capture (REDCap) system hosted at the University of Illinois Chicago.^29,30^ Visual acuity was measured at 3 m,^31^ right eye first, with presenting (habitual) correction and after refraction (best corrected), using an electronic visual acuity tester (Smart System 20/20, version 2010.1 [M&S Technologies]). Refraction and visual acuity testing followed electronic Early Treatment Diabetic Retinopathy Study (ETDRS)^31^ protocols after staff received Emmes OptymEdge ophthalmic certification (Emmes Corporation). Visual acuity was quantified as letters read correctly on an ETDRS eye chart and was also expressed as a logarithm of the minimum angle of resolution (logMAR) score.

Three VI outcomes^8,12,32^ were assessed in the better-seeing eye: VI by habitual visual acuity, by best-corrected visual acuity, and by uncorrected refractive error. VI was defined as visual acuity less than or equal to 20/40 (≤0.3 LogMAR) and blindness as less than 20/200 (<1.0 LogMAR), based on habitual and best-corrected measurements, per US standards.^1,9,14,33,34,35^ Uncorrected refractive error was defined as habitual visual acuity VI of 20/40 or worse improving to better than 20/40 with best correction.^12^ Outcomes addressed analytic aims assessing CVD-VI associations and NMDOH confounding in US Hispanic/Latino adults with high CVD and NMDOH burden vs World Health Organization VI definitions^36^ developed for worldwide surveillance.

### Individual and Cumulative Major CVD Risk Factors

The exposure was CVD risk factors (diabetes, hypercholesterolemia, obesity, smoking, and hypertension), defined using data collected at HCHS/SOL visit 3 and following national guidelines.^37,38,39,40^ The number of risk factors was assessed individually and cumulatively (0 or 1, 2, 3, or ≥4 risk factors),^19,41^ with more factors representing a more adverse CVD risk factor profile. Diabetes was classified using American Diabetes Association laboratory criteria (fasting plasma glucose level of ≥126 mg/dL or ≥200 mg/dL for ≤8 hours [to convert to mmol/L, multiply by 0.0555] or glycated hemoglobin level of ≥6.5% of total hemoglobin [to convert to proportion of total hemoglobin, multiply by 0.01]),^37^ self-reported diagnosis, or antihyperglycemic medication use.^20^ Hypercholesterolemia was defined as total cholesterol level 240 mg/dL or greater, low-density lipoprotein cholesterol level 160 mg/dL or greater, high-density lipoprotein cholesterol level less than 40 mg/dL, or cholesterol-lowering medication use (to convert cholesterol to mmol/L, multiply by 0.0259).^38^ Obesity was defined as a body mass index (BMI, calculated as weight in kilograms divided by height in meters squared) of 30.0 or greater.^40^ Smoking was defined as current cigarette use and having smoked more than 100 cigarettes over one’s lifetime. Hypertension was defined as systolic blood pressure of 130 mm Hg or higher, diastolic blood pressure of 80 mm Hg or higher, or self-reported antihypertensive medication use, per the American College of Cardiology and American Heart Association.^39^

### Covariates

Participants self-reported sociodemographic data, including Hispanic/Latino background, acculturation proxies, medical history, and medications. Backgrounds were Central American, Cuban, Mexican, Puerto Rican, South American, and other (included participants reporting multiple backgrounds; Dominican participants were grouped with this category to improve stability due to small sample size). Dietary intake was assessed using two 24-hour recalls.^19,42^ All data were collected at HCHS/SOL visit 3 except dietary intake (collected at visit 1). Details are provided in the eMethods in Supplement 1.

### Statistical Analysis

Sampling weights were calculated from the multistage design of HCHS/SOL and SOL Ojos for the Chicago and Miami field centers, adjusting for nonresponse (visit 1, visit 3, and SOL Ojos), to support inference for individuals in the original target populations and generate population-based estimates. Survey-specific procedures calculated estimates and 95% CIs. Estimates were age-standardized to the 2010 US Census population^43^ by age, sex, and Hispanic/Latino background (eMethods in Supplement 1), accounting for age distribution differences across Hispanic/Latino groups and the age-related nature of VI, ensuring internal validity. Age-standardized prevalence estimates for demographics, CVD risk factors, and VI outcomes were calculated by Hispanic/Latino group and sex. Age- and sex-adjusted prevalence of VI and cumulative CVD risk factors were internally adjusted to the SOL Ojos weighted mean (SD) age (58.7 [0.4] years) and sex proportion (50.2% [1.18%] female) for the VI analytic sample and were reported by NMDOH. Overall group significance was assessed using the Wald test; significant results were followed by pairwise comparisons based on 95% CI overlap to limit type I errors. Observations with missing non-VI data were excluded from relevant analyses (eg, for CVD risk factors, diabetes and hypertension had no missing observations, hypercholesterolemia and smoking had 1 missing observation each, and BMI had 5 missing observations; other variables had 0.03%-3.34% missingness).

Logistic regression assessed the association between CVD risk factors and VI, reported as odds ratios (ORs) and 95% CIs. Sequential modeling of risk factor sets^44^ evaluated whether NMDOH-related covariate adjustment attenuated associations using nested models. Model 1 adjusted for age, sex, Hispanic/Latino background, educational level, income, and marital status (traditional confounding). Model 2 additionally adjusted for all other CVD risk factors (diabetes, hypercholesterolemia, obesity, smoking, and hypertension [biologic confounding]). Model 3 further adjusted for nativity, health care access, and diet score (NMDOH-related contextual confounding relevant to Hispanic/Latino adults).

Models assessed changes in the association between CVD risk factors and VI attributable to traditional, biologic, and NMDOH-related confounding. Intermediate models evaluated changes in estimated ORs across adjustment stages, with statistical inference based on the fully adjusted final model. Changes in ORs were examined to assess attenuation with further adjustment. Age was modeled continuously; the other variables were categorical. Type III analysis with *F* tests compared final model results across cumulative CVD risk factors. Forest plots illustrated the outcomes of sequential adjustment.^45^ Statistical tests used a 2-sided significance level of *P* < .05, without adjustment for multiple comparisons given a priori, hypothesis-driven analysis of clinically relevant CVD risk factors; findings should be interpreted accordingly. Analyses were conducted using SAS, version 9.4 (SAS Institute Inc), at the SOL Ojos Coordinating Center, University of Illinois Chicago. Additional details are in the eMethods in Supplement 1.

## Results

Of 3864 eligible HCHS/SOL participants completing visit 3, SOL Ojos examined 3294 (85.2% overall; 1711 of 1922 individuals from Chicago [89.0%] and 1583 of 1942 [81.5%] from Miami). Six participants were excluded for missing VI data, leaving 3288 for analysis (mean age of the analytic sample, 58.7 years [95% CI, 57.9-59.5 years]; 61.9% [95% CI, 60.2%-63.5%] were female and 38.1% [95% CI, 36.5%-39.8%] were male). A total of 589 participants (14.4%) self-identified as Central American, 851 (42.3%) as Cuban, 1023 (21.6%) as Mexican, 324 (8.3%) as Puerto Rican, 400 (8.5%) as South American, and 111 (4.9%) as other background. Participant flow is shown in eFigure 1 in Supplement 1. Age-standardized demographics by background at visit 3 are in Table 1, with significant differences by background for all characteristics except marital status, income, resilience, and health care access.

**Table 1.** Age-Standardized Descriptive Characteristics of the SOL Ojos Study Population, by Hispanic/Latino Background

Characteristic	Proportion of participants, weighted % (95% CI)^a^							*P* value^c^
	All	Central American	Cuban	Mexican	Puerto Rican	South American	Other^b^	
Participants, No. (%)^d^	3288 (100)	589 (14.4)	851 (42.3)	1013 (21.6)	324 (8.3)	400 (8.5)	111 (4.9)	NA
Sex								
Men	50.0 (47.6-52.4)	43.4 (37.8-49.0)	54.0 (48.4-59.5)	50.0 (45.8-54.1)	56.4 (49.6-63.3)	38.6 (31.7-45.4)	47.7 (33.5-61.9)	.003
Women	50.0 (47.6-52.4)	56.6 (51.0-62.2)	46.0 (40.5-51.6)	50.0 (45.9-54.2)	43.6 (36.7-50.4)	61.4 (54.6-68.3)	52.3 (38.1-66.5)	
Standardized age, mean (95% CI), y	57.8 (57.7-58.0)	57.4 (57.1-57.8)	58.2 (58.0-58.5)	57.4 (57.2-57.7)	58.0 (57.4-58.5)	57.4 (57.0-57.8)	57.5 (56.6-58.4)	<.001
Marital status								
Single	19.2 (16.9-21.4)	26.2 (21.1-31.3)	17.3 (11.9-22.6)	13.4 (9.6-17.2)	27.0 (17.9-36.2)	19.0 (12.4-25.6)	23.0 (13.2-32.8)	.08
Married or living with partner	56.2 (53.4-59.0)	53.6 (47.7-59.4)	56.3 (51.1-61.4)	67.3 (62.8-71.9)	47.5 (38.4-56.5)	48.0 (39.2-56.7)	51.8 (38.9-64.6)	
Separated, divorced, or widowed	24.6 (22.5-26.8)	20.2 (15.6-24.9)	26.5 (21.9-31.1)	19.3 (15.9-22.7)	25.5 (17.2-33.8)	33.0 (25.9-40.1)	25.2 (14.8-35.7)	
Educational level								
<High school	27.3 (24.5-30.0)	35.6 (29.1-42.2)	19.5 (14.2-24.9)	47.2 (41.8-52.6)	29.0 (20.2-37.7)	13.7 (8.7-18.6)	20.3 (6.7-33.9)	<.001
High school graduate	25.3 (23.0-27.6)	23.0 (17.8-28.2)	24.1 (19.3-29.0)	29.8 (25.1-34.4)	24.9 (16.4-33.5)	21.5 (14.2-28.7)	15.3 (6.0-24.7)	
>High school	47.4 (44.4-50.4)	41.4 (36.2-46.6)	56.4 (49.6-63.1)	23.0 (19.2-26.9)	46.1 (36.5-55.7)	64.9 (56.4-73.4)	64.4 (50.5-78.2)	
Annual family income, $								
<10 000	13.0 (11.5-14.5)	13.1 (9.4-16.7)	15.0 (12.8-17.2)	6.2 (4.3-8.1)	11.7 (7.5-15.9)	9.3 (5.4-13.2)	13.9 (3.7-24.1)	.09
10 001-20 000	23.6 (21.0-26.2)	29.6 (24.4-34.8)	24.1 (17.8-30.4)	24.8 (21.1-28.4)	19.7 (13.5-25.9)	22.6 (16.9-28.2)	21.3 (11.3-31.3)	
20 001-40 000	30.5 (27.9-33.0)	33.8 (28.2-39.4)	25.0 (20.7-29.3)	36.8 (33.6-40.0)	26.6 (17.3-35.9)	34.4 (27.1-41.6)	19.6 (8.7-30.5)	
40 001-70 000	18.9 (16.3-21.4)	11.6 (8.0-15.3)	18.4 (12.3-24.4)	22.9 (19.0-26.7)	22.3 (14.5-30.2)	22.5 (16.8-28.2)	18.8 (9.1-28.5)	
>70 000	14.1 (11.7-16.4)	11.9 (7.1-16.8)	17.5 (11.6-23.5)	9.4 (6.7-12.1)	19.6 (11.7-27.5)	11.3 (6.4-16.2)	26.4 (12.2-40.6)	
Has health insurance	76.4 (73.5-79.3)	69.5 (65.0-73.9)	88.4 (83.3-93.5)	59.5 (55.1-63.9)	95.1 (89.4-100)	74.0 (65.9-82.0)	75.1 (63.3-86.8)	<.001
Nativity								
US born^e^	8.4 (6.3-10.4)	0.6 (0.0-1.5)	9.4 (3.4-15.4)	5.4 (3.1-7.7)	36.2 (27.2-45.3)	2.1 (0.0-4.3)	25.6 (14.4-36.8)	<.001
Not US born								
≥20 y in US	61.3 (57.9-64.7)	77.1 (71.7-82.5)	42.0 (36.7-47.3)	81.5 (77.2-85.7)	59.7 (50.3-69.1)	61.3 (53.4-69.2)	50.5 (39.0-62.1)	
<20 y in US	30.3 (26.8-33.8)	22.3 (16.9-27.7)	48.6 (41.5-55.8)	13.2 (9.3-17.1)	4.1 (1.0-7.3)	36.7 (28.6-44.7)	23.9 (10.5-37.3)	
Immigrant generation								
First	91.9 (89.9-94.0)	99.3 (98.3-100)	90.5 (84.4-96.6)	94.9 (92.5-97.4)	66.4 (56.7-76.0)	97.8 (95.3-100)	75.0 (63.4-86.5)	<.001
Second or higher	8.1 (6.0-10.1)	0.7 (0.0-1.7)	9.5 (3.4-15.6)	5.1 (2.6-7.5)	33.6 (24.0-43.3)	2.2 (0.0-4.7)	25.0 (13.5-36.6)	
Language preference								
English	8.5 (6.4-10.5)	3.5 (0.8-6.2)	7.7 (2.0-13.4)	5.7 (3.4-8.1)	44.5 (34.7-54.3)	3.4 (0.7-6.1)	12.7 (4.3-21.0)	<.001
Spanish	91.5 (89.5-93.6)	96.5 (93.8-99.2)	92.3 (86.6-98.0)	94.3 (91.9-96.6)	55.5 (45.7-65.3)	96.6 (93.9-99.3)	87.3 (79.0-95.7)	
Stress of Immigration Scale score, mean (95% CI)^f^	1.5 (1.5-1.5)	1.7 (1.6-1.8)	1.4 (1.3-1.4)	1.6 (1.6-1.7)	1.2 (1.1-1.2)	1.6 (1.5-1.8)	1.3 (1.2-1.4)	<.001
Chronic stress burden score, mean (95% CI)^g^	0.8 (0.8-0.9)	0.8 (0.7-0.9)	0.7 (0.6-0.8)	0.7 (0.6-0.8)	1.6 (1.3-1.8)	1.0 (0.8-1.1)	0.8 (0.5-1.1)	<.001
Brief Resilience Scale score, mean (95% CI)^h^	2.5 (2.5-2.5)	2.5 (2.4-2.5)	2.5 (2.5-2.6)	2.5 (2.5-2.6)	2.5 (2.4-2.6)	2.4 (2.3-2.5)	2.3 (2.1-2.5)	.14
Unable to access health care in past 12 mo	3.9 (3.0-4.8)	4.4 (2.8-6.0)	2.4 (1.2-3.5)	3.4 (2.1-4.6)	6.2 (2.0-10.4)	4.4 (1.9-7.0)	11.2 (1.7-20.7)	.18
Healthy diet score^i^	53.9 (50.6-57.3)	50.2 (44.6-55.7)	42.5 (36.8-48.2)	80.0 (76.0-84.0)	41.0 (30.2-51.8)	53.8 (46.4-61.2)	63.8 (49.6-78.0)	<.001

Abbreviations: NA, not applicable; SOL, Study of Latinos.

^a^ Values except for number of participants were weighted for study design and nonresponse and age standardized to the 2010 US Census population.

^b^ Due to small sample size, Dominican participants were grouped in the “other” category, which also included participants reporting multiple backgrounds, to improve stability.

^c^ *P* value based on Wald tests across all background groups.

^d^ Represents the number of participants with nonmissing values for Hispanic/Latino background.

^e^ Includes the 50 US states and Washington, DC only.

^f^ Range, 1 to 5; higher scores indicate more stress.

^g^ Range, 0 to 8; higher scores indicate increased stress.

^h^ Range, 1 to 5; higher scores indicate more resilience to recover from stress.

^i^ See eMethods in Supplement 1.

### Prevalence of Individual and Cumulative Major CVD Risk Factors

**Figure 1.**
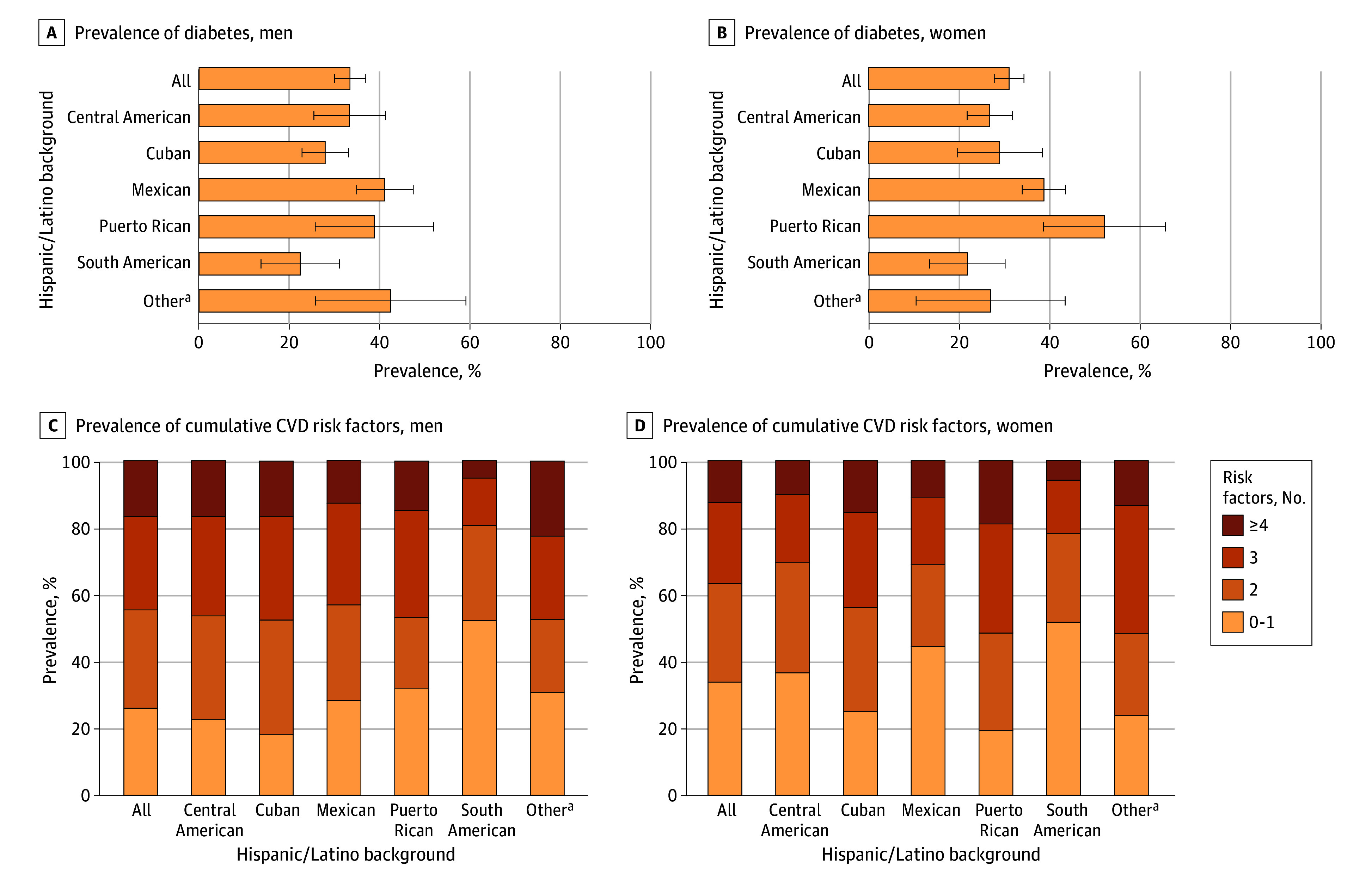
Bar Graphs Showing Age-Standardized Prevalence of Cardiovascular Disease (CVD) Risk Factors at Visit 3 in the SOL Ojos Study Population, by Hispanic/Latino Background Estimates were weighted for study design and nonresponse and age-standardized to the 2010 US Census population. eTable 1 in Supplement 1 presents the tabular data used to generate the graphs. A, B, Whiskers indicate 95% CIs. ^a^Other backgrounds included participants reporting multiple backgrounds; Dominican participants were also grouped in this category to improve stability.

Figure 1 and eTable 1 and eFigure 2 in Supplement 1 present age-standardized prevalence of major CVD risk factors at visit 3: diabetes (men, 33.4% [95% CI, 30.0%-36.9%]; women, 31.1% [95% CI, 27.8%-34.4%]), hypercholesterolemia (men, 59.0% [95% CI, 54.9%-63.1%]; women, 47.9% [95% CI, 44.4%-51.4%]), hypertension (men, 73.7% [95% CI, 69.9%-77.5%]; women, 65.0% [95% CI, 61.4%-68.6%]), obesity (men, 44.3% [95% CI, 39.7%-48.8%]; women, 51.2% [95% CI, 47.9%-54.4%]), and smoking (men, 21.4% [95% CI, 18.0%-24.8%]; women, 10.7% [95% CI, 7.9%-13.5%]). Prevalence varied by background for all risk factors except hypercholesterolemia in men. For example, diabetes was more common in Mexican than South American men (41.1% [95% CI, 34.9%-47.4%] vs 22.4% [95% CI, 13.7%-31.1%]) and women (38.8% [95% CI, 34.0%-43.6%] vs 21.9% [95% CI, 13.5%-30.2%]). Puerto Rican women had the highest diabetes prevalence (52.2% [95% CI, 38.7%-65.7%]). Prevalence of 1 or no CVD risk factors was lower than prevalence of 3 or more risk factors, with significant variation across backgrounds in both sexes. Full details are in Figure 1 and eTable 1 and eFigure 2 in Supplement 1.

### Prevalence of VI Outcomes

Table 2 presents age-standardized prevalence of VI. No sex differences were observed for VI prevalence assessed by habitual visual acuity overall (men, 7.1% [95% CI, 5.3%-8.8%]; women, 7.0% [95% CI, 5.2%-8.8%]), best-corrected visual acuity (men, 1.8% [95% CI, 1.0%-2.6%]; women, 2.6% [95% CI, 1.6%-3.7%]), or uncorrected refractive error (men, 5.2% [95% CI, 3.7%-6.8%]; women, 4.4% [95% CI, 3.0%-5.8%]).

**Table 2.** Age-Standardized Prevalence of Visual Impairment in the SOL Ojos Study Population by Hispanic/Latino Group^a^

Characteristic	Proportion of participants with VI, weighted % (95% CI)^b^							*P* value^d^
	All	Central American	Cuban	Mexican	Puerto Rican	South American	Other^c^	
Participants, No. (%)^e^								
All	3288 (100)	589 (14.4)	851 (42.3)	1013 (21.6)	324 (8.3)	400 (8.5)	111 (4.9)	NA
Men	1254 (50.0)	190 (43.4)	364 (54.0)	392 (50.0)	124 (56.4)	140 (38.6)	44 (47.7)	.003
Women	2034 (50.0)	399 (56.6)	487 (46.0)	621 (50.0)	200 (43.63)	260 (61.4)	67 (52.3)	
Better-eye habitual visual acuity correction^f^								
Men	7.1 (5.3-8.8)	9.2 (2.9-15.4)	4.1 (1.8-6.4)	16.9 (11.6-22.2)	4.7 (2.1-7.4)	6.9 (2.0-11.8)	8.6 (1.7-15.6)	.001
Women	7.0 (5.2-8.8)	5.2 (2.3-8.1)	5.3 (3.1-7.5)	11.1 (7.9-14.3)	7.9 (3.6-12.2)	6.3 (2.2-10.4)	9.9 (0.0-22.7)	.08
Better-eye best-corrected visual acuity^f^								
Men	1.8 (1.0-2.6)	2.3 (0.0-5.8)	1.5 (0.0-3.3)	3.4 (1.5-5.4)	2.4 (0.2-4.6)	3.4 (0.0-7.3)	0.0 (0.0-0.0)	<.001
Women	2.6 (1.6-3.7)	0.8 (0.2-1.4)	2.3 (0.7-4.0)	4.5 (2.6-6.5)	3.2 (0.1-6.3)	3.0 (0.2-5.7)	0.6 (0.0-1.8)	.001
Better-eye uncorrected refractive error^g^								
Men	5.2 (3.7-6.8)	6.8 (2.0-11.7)	2.6 (1.1-4.1)	13.5 (8.1-18.9)	2.3 (0.6-4.0)	3.6 (0.4-6.7)	8.6 (1.7-15.6)	.006
Women	4.4 (3.0-5.8)	4.4 (1.6-7.3)	2.9 (1.5-4.3)	6.6 (4.3-8.8)	4.7 (1.7-7.7)	3.3 (1.2-5.4)	9.3 (0.0-22.0)	.11

Abbreviations: NA, not applicable; SOL, Study of Latinos.

^a^ Visual impairment was defined as visual acuity less than or equal to 20/40.

^b^ Values except for numbers of participants were weighted for study design and nonresponse and age-standardized to the 2010 US Census population. Prevalence of visual impairment and uncorrected refractive error are reported at the person level according to the better-seeing eye.

^c^ Due to small sample size, Dominican participants were grouped in the “other” category, which also included participants reporting multiple backgrounds, to improve stability.

^d^ *P* values were based on Wald tests across all background groups.

^e^ Represents the number of participants with nonmissing values for Hispanic/Latino background. Six of 3294 participants examined at visit 3 of the Hispanic Community Health Study/Study of Latinos had missing background data and were excluded from analyses.

^f^ Participants with habitual visual acuity worse than 20/200 (n = 10) or best-corrected visual acuity worse than 20/200 (n = 8) were grouped into visual impairment assessed by habitual visual acuity or by best-corrected visual acuity, respectively, due to small cell size.

^g^ Uncorrected refractive error was defined as habitual visual acuity equal to or worse than 20/40 that improved to best-corrected visual acuity better than 20/40.

Significant variation (Wald test) according to Hispanic/Latino background was observed for VI prevalence assessed by habitual visual acuity and uncorrected refractive error in men (Table 2). VI prevalence varied across Hispanic/Latino backgrounds in men (range by habitual visual acuity, 4.1% [95% CI, 1.8%-6.4%] to 16.9% [95% CI, 11.6%-22.2%] and by uncorrected refractive error, 2.3% [95% CI, 0.6%-4.0%] to 13.5% [95% CI, 8.1%-18.9%]), and highest prevalence using both measures was among men identifying as Mexican. Minimal pairwise differences in VI by best-corrected visual acuity should be interpreted cautiously given near-0 prevalence and small sample sizes, yielding unstable estimates.

### Prevalence of VI Outcomes and Cumulative CVD Risk Factors by NMDOH

Table 3 reports age- and sex-adjusted prevalence of VI outcomes and cumulative CVD risk factor profiles by NMDOH. VI prevalence increased with age and was highest in individuals aged 80 years or older and lowest in those aged 40 to 49 years. Higher VI prevalence was associated with lower socioeconomic status, including lower educational level, lower income, Spanish vs English language preference, and limited health care access (detailed results are in Table 3).

**Table 3.** Age- and Sex-Adjusted Prevalence of Adverse CVD Risk Factors, Visual Impairment, and URE in the SOL Ojos Study Population by Various NMDOH

Characteristic	Prevalence of VI, % (95% CI)^a^		URE, % (95% CI)^e^	Prevalence of CVD risk factors, % (95% CI)^b^			
	Habitual visual acuity^c^	Best-corrected visual acuity^d^		0 or 1	2	3	≥4
Age group, y							
40-49	3.6 (2.1-6.2)^f^	0.4 (0.1-1.9)^f^	3.2 (1.7-5.8)^g^	44.3 (37.8-50.8)^f^	29.2 (24.3-34.2)	18.7 (13.0-24.4)^f^	7.7 (3.9-11.5)^g^
50-59	4.1 (2.4-6.7)	1.0 (0.2-4.1)	3.1 (2.0-4.7)	29.3 (24.7-33.9)	29.3 (25.0-33.5)	24.9 (20.6-29.3)	16.5 (13.1-19.9)
60-69	8.8 (7.0-11.1)	2.3 (1.4-3.7)	6.5 (5.0-8.4)	20.8 (17.8-23.8)	31.9 (28.5-35.4)	30.7 (27.2-34.3)	16.6 (14.0-19.1)
70-79	11.9 (9.0-15.4)	3.9 (2.4-6.0)	7.9 (5.5-11.4)	16.4 (12.0-20.8)	26.0 (21.0-30.9)	35.3 (29.7-41.0)	22.3 (17.5-27.1)
≥80	22.4 (14.2-33.5)	13.8 (7.7-23.5)	8.0 (3.7-16.5)	16.4 (8.2-24.7)	34.4 (23.7-45.2)	33.8 (23.2-44.4)	15.4 (6.4-24.3)
Sex							
Men	7.3 (5.6-9.5)	1.3 (0.6-2.8)	5.6 (4.2-7.5)	21.4 (17.8-24.9)^g^	30.2 (26.5-33.9)	30.1 (26.5-33.8)	18.3 (15.0-21.5)^h^
Women	7.0 (5.2-9.3)	1.8 (1.1-3.1)	4.5 (3.2-6.2)	28.9 (25.5-32.2)	31.2 (27.8-34.6)	26.4 (23.0-29.9)	13.5 (11.2-15.8)
Educational level							
<High school	10.6 (8.1-13.8)^f^	2.9 (1.3-6.3)^g^	6.4 (4.8-8.5)	21.9 (18.1-25.6)	30.6 (26.5-34.7)	31.6 (26.3-36.8)	16.0 (12.5-19.5)
High school graduate	7.4 (5.1-10.7)	1.5 (0.7-3.1)	5.5 (3.6-8.5)	24.7 (20.2-29.2)	30.0 (24.8-35.1)	28.8 (24.2-33.5)	16.5 (11.5-21.5)
>High school	4.9 (3.5-6.9)	0.9 (0.4-1.8)	3.9 (2.7-5.6)	27.0 (22.9-31.2)	31.5 (27.8-35.3)	26.1 (22.0-30.2)	15.3 (12.7-18.0)
Annual family income, $							
<10 000	7.7 (5.1-11.5)	2.2 (0.8-5.7)	4.7 (2.9-7.6)^h^	21.9 (15.3-28.5)^g^	30.3 (23.6-37.0)	27.7 (21.1-34.4)	20.0 (12.6-27.4)^h^
10 001-20 000	9.5 (6.9-13.0)	1.8 (0.6-5.3)	7.5 (5.3-10.3)	19.3 (15.1-23.4)	31.1 (26.6-35.6)	30.7 (25.0-36.4)	18.9 (15.0-22.7)
20 001-40 000	6.1 (4.5-8.2)	1.9 (1.1-3.3)	3.8 (2.7-5.3)	28.5 (23.7-33.2)	29.7 (25.2-34.2)	28.4 (23.6-33.1)	13.5 (10.1-16.8)
40 001-70 000	5.9 (3.8-9.2)	1.1 (0.5-2.4)	4.4 (2.6-7.5)	26.7 (21.2-32.2)	35.8 (29.9-41.6)	24.8 (18.9-30.8)	12.7 (8.0-17.4)
>70 000	3.5 (1.2-10.2)	0.3 (0.0-2.1)	2.9 (0.9-9.1)	30.6 (22.9-38.2)	29.8 (21.7-37.8)	28.3 (19.8-36.9)	11.3 (7.5-15.2)
Marital status							
Single	9.1 (6.0-13.6)	1.8 (0.7-4.5)	6.5 (4.0-10.4)	23.5 (18.0-29.0)	27.7 (21.6-33.8)	30.1 (23.6-36.5)	18.8 (12.4-25.1)
Separated, divorced, or widowed	7.1 (4.9-10.1)	1.4 (0.7-2.8)	5.1 (3.2-8.1)	22.0 (17.5-26.6)	29.5 (25.0-34.0)	31.5 (25.4-37.5)	17.0 (13.0-21.0)
Married or living with partner	6.5 (5.1-8.3)	1.6 (0.8-3.0)	4.4 (3.3-5.9)	27.0 (23.8-30.2)	32.6 (29.0-36.2)	26.2 (23.1-29.3)	14.2 (11.8-16.6)
Health insurance							
No	9.7 (6.6-14.1)	1.4 (0.6-3.4)	7.3 (4.8-10.9)	29.2 (23.4-35.1)	31.9 (26.2-37.7)	24.4 (17.8-31.0)	14.4 (9.7-19.2)
Yes	6.6 (5.0-8.6)	1.6 (0.8-3.3)	4.5 (3.3-6.0)	24.1 (21.2-27.0)	30.5 (27.7-33.4)	29.3 (26.4-32.2)	16.1 (14.0-18.2)
Nativity							
US born^i^	5.5 (2.6-11.6)	0.8 (0.2-3.1)	4.1 (1.7-9.4)	22.7 (13.4-31.9)	21.1 (11.4-30.7)	38.6 (24.7-52.5)	17.7 (9.5-25.9)
Not US born							
≥20 y in US	8.1 (6.4-10.4)	1.8 (0.9-3.4)	5.7 (4.4-7.4)	24.0 (20.9-27.0)	32.9 (29.6-36.1)	26.8 (23.8-29.7)	16.4 (13.8-19.0)
<20 y in US	5.4 (3.5-8.1)	1.3 (0.5-2.9)	3.7 (2.2-6.0)	27.7 (22.3-33.2)	29.2 (24.7-33.8)	29.6 (24.0-35.2)	13.4 (10.4-16.5)
Immigrant generation							
First	7.1 (5.7-8.8)	1.3 (0.7-2.2)	5.1 (4.0-6.5)	25.3 (22.4-28.2)	31.8 (29.0-34.5)	27.4 (24.7-30.2)	15.5 (13.4-17.5)
≥Second	6.2 (2.9-13.0)	0.8 (0.2-3.4)	4.5 (1.9-10.4)	19.6 (10.0-29.1)	23.1 (12.5-33.7)	37.9 (22.6-53.2)	19.4 (10.4-28.4)
Language preference							
English	3.5 (1.9-6.4)^h^	1.8 (0.6-5.4)	1.6 (0.8-3.0)^f^	19.8 (11.5-28.1)	25.9 (15.6-36.2)	32.6 (19.0-46.1)	21.7 (11.5-31.9)
Spanish	7.4 (6.0-9.2)	1.6 (0.8-2.9)	5.3 (4.2-6.6)	25.4 (22.6-28.2)	31.2 (28.7-33.8)	28.0 (25.4-30.6)	15.3 (13.2-17.5)
Able to access health care in past 12 mo							
No	11.9 (6.1-21.9)	4.4 (1.8-10.5)^h^	6.3 (2.5-15.0)	13.9 (7.2-20.6)^h^	33.3 (20.1-46.5)	27.5 (17.1-38.0)	25.3 (12.4-38.1)
Yes	7.0 (5.6-8.7)	1.5 (0.8-2.7)	4.9 (3.9-6.2)	25.3 (22.5-28.0)	30.7 (28.1-33.3)	28.6 (25.8-31.3)	15.5 (13.5-17.5)
Healthy diet score^j^							
Lower 60%	6.8 (4.9-9.2)	1.6 (0.9-2.9)	4.8 (3.5-6.7)	21.7 (18.3-25.1)^g^	31.8 (28.0-35.6)	29.9 (25.8-34.0)	16.6 (13.5-19.6)
Higher 40%	7.3 (5.7-9.3)	1.5 (0.7-3.1)	4.9 (3.7-6.5)	27.8 (24.4-31.2)	29.9 (26.0-33.7)	27.0 (23.4-30.5)	15.4 (12.5-18.2)

Abbreviations: CVD, cardiovascular disease; NMDOH, nonmedical drivers of health; SOL, Study of Latinos; URE, uncorrected refractive error.

^a^ Values weighted for study design and nonresponse. Prevalence by age was adjusted for sex; prevalence by sex was adjusted for age.

^b^ See Individual and Cumulative Major CVD Risk Factors in the Methods.

^c^ Less than or equal to 20/40 in the better-seeing eye, with presenting (habitual) correction.

^d^ Worse than or equal to 20/40 in the better-seeing eye after refraction.

^e^ Worse than or equal to 20/40 that improved to better than 20/40 best-corrected visual acuity in the better-seeing eye.

^f^ Wald test *P* < .001.

^g^ Wald test *P* < .01.

^h^ Wald test *P* < .05.

^i^ Fifty US states and Washington, DC only.

^j^ See eMethods in Supplement 1.

### ORs for VI Prevalence by Individual and Cumulative CVD Risk Factors

**Figure 2.**
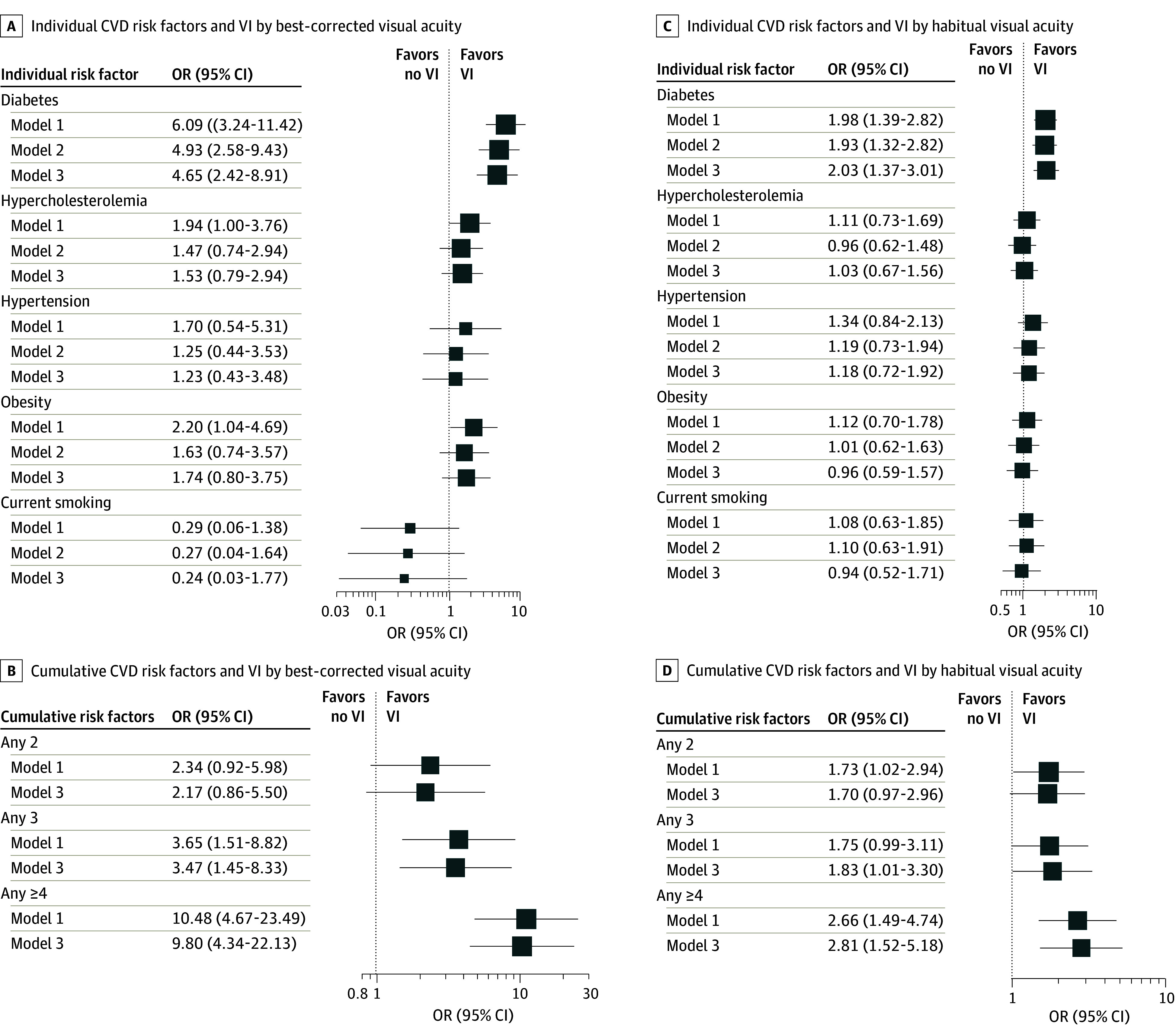
Forest Plots of Odds Ratio (OR) Estimates for Visual Impairment (VI) Associated With Major Cardiovascular Disease (CVD) Risk Factors in the SOL Ojos Study Population, by Habitual and Best-Corrected Visual Acuity The ORs and 95% CIs were from nested, sequential modeling. eTable 2 in Supplement 1 presents the tabular data used to generate the forest plots. Larger data markers indicate greater precision. Model 1 adjusted for age, sex, Hispanic/Latino background, educational level, income, and marital status (traditional confounding). Model 2 adjusted for factors in model 1 plus all other CVD risk factors (biologic confounding—individual CVD risk factor exposures due only to collinearity with cumulative CVD risk factors). Model 3 adjusted for factors in model 2 plus diet, health care access, and nativity (contextual confounding related to nonmedical drivers of health relevant to Hispanic/Latino adults—thus, adjusted for all risk factor categories).

Figure 2 and eTables 2 and 3 in Supplement 1 present adjusted logistic regression results for associations between individual and cumulative CVD risk factors and VI outcomes, with models for traditional (model 1), biologic (model 2), and contextual (model 3) confounders. For individual CVD risk factors (Figure 2A and C), diabetes was associated with VI assessed by habitual visual acuity (model 3 OR, 2.03 [95% CI, 1.37-3.01]) and by best-corrected visual acuity (model 3 OR, 4.65 [95% CI, 2.42-8.91]). In individuals with diabetes, ORs for VI by best-corrected visual acuity were attenuated in model 2 (biologic), reducing from 6.09 (95% CI, 3.24-11.42) in the traditional model to 4.93 (95% CI, 2.58-9.43) (reduction of 19.0%), and in model 3 (contextual), reducing from 4.93 (95% CI, 2.58-9.43) in the biologic model to 4.65 (95% CI, 2.42-8.91) (reduction of 5.7%), while ORs for VI by habitual visual acuity remained stable across models. There was no association between diabetes and VI assessed by uncorrected refractive error (model 3: OR, 1.36 [95% CI, 0.85-2.20]) (eFigure 3 and eTables 2 and 3 in Supplement 1).

For cumulative CVD risk factors (Figure 2B and D), having more risk factors was associated with higher odds of VI assessed by habitual and best-corrected visual acuity. By habitual visual acuity, the OR for VI in individuals with 2 risk factors in model 3 was 1.70 (95% CI, 0.97-2.96), with 3 risk factors was 1.83 (95% CI, 1.01-3.30), and with 4 or more risk factors was 2.81 (95% CI, 1.52-5.18) compared with 1 or 0 risk factors (in type III analysis with *F* test, all *P* < .05). By best-corrected visual acuity, ORs for VI were 3.47 (95% CI, 1.45-8.33) in individuals with 3 risk factors and 9.80 (95% CI, 4.34-22.13) with 4 or more risk factors compared with 1 or 0 risk factors (type III analysis with *F* test, both *P* < .001). Minimal attenuation across models suggested limited contextual confounding. Associations with uncorrected refractive error were near null (eFigure 3 and eTables 2 and 3 in Supplement 1).

## Discussion

SOL Ojos revealed age-standardized VI prevalence across US Hispanic/Latino groups comprising the largest minority population in the US, the growth of which is the largest contributor to growth of the US population overall.^16^ We assessed VI outcomes by 3 different measures: (1) habitual visual acuity, reflecting clinical impairment from inadequate correction and ocular pathology; (2) best-corrected visual acuity, reflecting impairment from pathology; and (3) uncorrected refractive error, reflecting impairment treatable with corrective eyewear. Given shared risk factors between chronic eye disease and CVD, we examined associations between individual and cumulative CVD risk factors and VI. We examined attenuation of the CVD-VI association by NMDOH, sequentially adjusting for traditional, biologic, and NMDOH-contextual factors. This approach evaluated confounders and compared patterns across VI outcomes by all measures, highlighting how VI manifests in a population at high CVD risk with NMDOH barriers.

The first key finding was VI outcome heterogeneity across Hispanic/Latino backgrounds. Men of Mexican background had more than 4-fold higher prevalence of VI assessed by habitual visual acuity and nearly 6-fold higher prevalence of VI assessed by uncorrected refractive error than the lowest-prevalence groups. This highlights the need to disaggregate Hispanic/Latino groups in research.^46,47^ These differences may reflect such things as variation in cultural factors, health care access and literacy, migration patterns, occupational exposures, and broader structural factors affecting CVD risk and eye care access.

Second, having vs not having diabetes was associated with different VI outcomes: 2-fold higher odds of VI assessed by habitual visual acuity, more than 4-fold higher odds of VI assessed by best-corrected visual acuity, and no association with VI assessed by uncorrected refractive error. When uncorrected refractive error was excluded, heterogeneity was reduced and higher odds of VI in patients with diabetes were found, consistent with pathophysiology and conceptual distinctions among VI outcomes. This mirrors meta-analysis findings that showed larger effect sizes for the association of VI with all-cause mortality when VI was assessed by best-corrected rather than presenting (habitual) visual acuity.^48^

Third, having 3 or more cumulative CVD risk factors was associated with VI assessed by habitual visual acuity and by best-corrected visual acuity, with highest odds for VI assessed by best-corrected visual acuity among individuals with 4 or more risk factors. No significant associations were observed for uncorrected refractive error. Diabetes and high CVD burden were each associated with VI assessed by best-corrected visual acuity, emphasizing the need for careful outcome-specific assessment in high-risk populations. Although etiologies were not assessed, best-corrected visual acuity likely reflects chronic eye diseases (eg, diabetic retinopathy, age-related macular degeneration, glaucoma, or cataract), consistent with its associations with diabetes and cumulative CVD burden.

Fourth, sequential modeling showed minimal NMDOH-related contextual confounding in CVD-VI associations but greater biologic confounding (eg, 19.0% reduction in odds of VI by best-corrected visual acuity in the biologic vs traditional model in individuals with diabetes), highlighting the need to adjust for coexisting CVD risk factors. After full adjustment, diabetes remained associated with VI assessed by best-corrected visual acuity (OR, 4.65 [95% CI, 2.42-8.91]). NMDOH minimally attenuated CVD-VI associations, but they may act as upstream determinants (influencing CVD risk factors associated with VI) or mediators or they may remain incompletely captured, with structural barriers such as health care access, socioeconomic constraints, and inequities influencing CVD and/or VI.

Fifth, individual and cumulative CVD risk factors varied significantly across Hispanic/Latino groups, likely reflecting baseline differences (2008-2011)^20,21,22,23,24,25,26^ and age-related progression. High CVD burden, particularly having diabetes or 4 or more risk factors, was associated with higher VI prevalence assessed by best-corrected visual acuity. Given the widespread prevalence of modifiable CVD risk factors (well-established in CVD prevention^49,50^ but less considered in preventive eye care), these factors associated with VI may offer public health benefits and opportunities for longitudinal research on incident VI.

Sixth, SOL Ojos identified associations between traditional NMDOH risk factors and VI,^8,9,10,11,12,32,33,34,51,52,53,54^ especially VI assessed by habitual visual acuity and uncorrected refractive error. Higher VI prevalence was observed among individuals with limited health care access, lower socioeconomic status, older age, and Spanish language preference. These findings align with results from the American Academy of Ophthalmology’s Intelligent Research in Sight (IRIS) Registry,^55^ which identified 2 key findings in Hispanic patients: underrepresentation in ophthalmology practices and 87% higher likelihood of corrected VI (<20/40) than in non-Hispanic White individuals.^52^ Addressing VI may include expanding eye care access, providing culturally appropriate education, implementing community-based vision screening, and improving ophthalmology referral pathways.

SOL Ojos revealed a higher prevalence of VI assessed by habitual visual acuity (approximately 7%) and uncorrected refractive error (approximately 4%-5%) than by best-corrected visual acuity (approximately 2%), highlighting eye care underutilization, consistent with IRIS registry findings.^52^ Uncorrected refractive error, a leading cause of VI,^8,9,10,11,12,13,14,15^ represents an opportunity to address adverse outcomes through corrective eyewear. VI is associated with cognitive decline,^2,3^ mobility loss,^4^ depression,^5^ and all-cause mortality,^6^ especially when occurring with newly diagnosed type 2 diabetes^7^; furthermore, even mild VI is associated with unemployment and underemployment.^56,57,58^ VI assessed by habitual visual acuity in disadvantaged populations captures chronic visual challenges signaling elevated risk beyond VI assessed by best-corrected visual acuity. Men of Mexican background have been found to exhibit the highest prevalence, suggesting elevated risk for systemic adverse outcomes.^2,3,4,5,6,7,56,57,58^ Given the high overall VI burden and subgroup differences in VI prevalence in this study, expanding access to corrective eyewear could address VI and these previously associated health risks.

Our findings emphasize the value of population-based research with unbiased prevalence estimates. SOL Ojos used gold-standard ETDRS protocols,^31^ yielding an approximate 2% VI prevalence assessed by best-corrected visual acuity, consistent with prevalence among Hispanic/Latino persons in a meta-analysis.^1^ In contrast, IRIS registry estimates for Hispanic individuals were higher (10%),^52^ likely reflecting biases in datasets restricted to care-seeking individuals. Other US population-based studies and national surveys corroborate SOL Ojos findings (overlapping 95% CIs),^8,9,10,11,51,59^ but differences in age distributions, lack of external age standardization^43^ and reliance on age-stratified^8,9,12,33,34^ or internally age-adjusted results,^12,33,34^ and differences in VI thresholds^8,11,12,51,54,59^ limit direct comparability. However, associations of traditional NMDOH factors in our study, including older age,^8,9,10,11,12,32,33,34,51,52^ lower socioeconomic status,^8,9,11,32,51,53^ minority race and ethnicity,^10,12,32,51,52,54^ and limited health care access or insurance,^8,9,11,51,52^ were consistent with prior research. The relationship between CVD factors and prevalent VI remains less defined, with some studies identifying diabetes as a risk factor^9,11^ and others showing inconsistent associations.^11,51^ In this US cohort of Hispanic/Latino individuals with high CVD burden, detailed cardiovascular phenotyping revealed associations of individual and cumulative^19,41^ CVD risk factors with prevalence of VI, likely obscured in lower-burden or less-phenotyped cohorts, extending Singapore Epidemiology of Eye Diseases (SEED) study findings.^60^

### Strengths and Limitations

This study has strengths. SOL Ojos leverages its multicenter design within the highly phenotyped HCHS/SOL cohort of distinct Hispanic/Latino groups.^17,18^ Data were weighted for sampling design, ensuring accurate population-level estimates. Participation exceeded 85%, surpassing recruitment targets by 10%, with ETDRS protocols^31^ providing more reliable estimates than self-reported or clinical data. Age standardization to the 2010 US Census^43^ ensured internal validity across Hispanic/Latino groups and external generalizability. SOL Ojos protocols aligned with prior cohorts on VI,^1,9,14,33,34,53^ individual^37,38,39,40^ and multiple^19,41^ CVD risk factors, and national surveys.^61,62,63,64^

Several limitations also warrant consideration. Low VI prevalence (approximately 2%-7%) limited statistical power, especially within subgroups, although dose-response trends supported the findings. The cross-sectional design limits causal inference, and CVD-VI associations may be bidirectional; however, the upcoming HCHS/SOL visit 4 (2027-2030) offers SOL Ojos longitudinal opportunities. Healthy volunteer bias may have underrepresented individuals with VI and mobility issues, underestimating prevalence. Baseline diet patterns (2008-2011) may not reflect those at visit 3 (2020-2024), potentially affecting CVD-VI associations, as diet score is an NMDOH confounder. Residual confounding remains possible despite adjustments.

## Conclusions

In this cross-sectional study of diverse US Hispanic/Latino adults with elevated CVD burden, SOL Ojos identified significant variation in VI prevalence, with the highest VI prevalence ascertained by habitual visual acuity and uncorrected refractive error in Mexican men. Diabetes and cumulative CVD risk factors were associated with VI assessed by best-corrected visual acuity. While SOL Ojos provides timely insights into the prevalence of VI, its cross-sectional design and findings underscore the need for longitudinal studies to examine incident disease and refine causal pathways.

## Notes

Supplement 1.
eMethods.

eReferences.
**eFigure 1.** Strengthening the Reporting of Observational Studies in Epidemiology (STROBE) Diagram: Hispanic Community Health Study/Study of Latinos (HCHC/SOL) and SOL Ojos Participant Flow **eFigure 2.** Age-Standardized Prevalence of Major Cardiovascular Disease Risk Factors in SOL Ojos Study Population, by Hispanic/Latino Background **eFigure 3.** Odds Ratios Estimates Between Major Cardiovascular Disease Risk Factors and Uncorrected Refractive Error in the SOL Ojos Study Population **eTable 1.** Tabular Data Used to Generate Figure 1 and eFigure 2: Age-Standardized Prevalence and 95% CIs of Cardiovascular Disease Risk Factors of the SOL Ojos Study Population, by Hispanic/Latino Background **eTable 2.** Tabular Data used to Generate Figure 2 and eFigure 3: Odds Ratio Estimates and 95% CIs of Visual Impairment in SOL Ojos Study Population **eTable 3.** Final Model Estimates (Model 3) Used to Generate Figure 2 and eFigure 3: Odds Ratio Estimates and 95% CIs between Individual and Cumulative Cardiovascular Disease Risk Factors and Visual Impairment in SOL Ojos Population

Supplement 2.
Members of the SOL Ojos Study Group

Supplement 3.
Data Sharing Statement
