# Supplementary material for: Visual Impairment and Cardiovascular Risk Factors in Hispanic and Latino Adults
Source: JAMA Netw Open. 2026 Jun 12;9(6):e2617975. doi: 10.1001/jamanetworkopen.2026.17975 (PMC13263780; doi:10.1001/jamanetworkopen.2026.17975)
Supplement: Supplement 1. — eMethods. eReferences. eFigure 1. Strengthening the Reporting of Observational Studies in Epidemiology (STROBE) Diagram: Hispanic Community Health Study/Study of Latinos (HCHC/SOL) and SOL Ojos Participant Flow eFigure 2. Age-Standardized Prevalence of Major Cardiovascular Disease Risk Factors in SOL Ojos Study Population, by Hispanic/Latino Background eFigure 3. Odds Ratios Estimates Between Major Cardiovascular Disease Risk Factors and Uncorrected Refractive Error in the SOL Ojos Study Population eTable 1. Tabular Data Used to Generate Figure 1 and eFigure 2: Age-Standardized Prevalence and 95% CIs of Cardiovascular Disease Risk Factors of the SOL Ojos Study Population, by Hispanic/Latino Background eTable 2. Tabular Data used to Generate Figure 2 and eFigure 3: Odds Ratio Estimates and 95% CIs of Visual Impairment in SOL Ojos Study Population eTable 3. Final Model Estimates (Model 3) Used to Generate Figure 2 and eFigure 3: Odds Ratio Estimates and 95% CIs between Individual and Cumulative Cardiovascular Disease Risk Factors and Visual Impairment in SOL Ojos Population [file jamanetwopen-e2617975-s001.pdf]

Supplementary Online Content

Joslin CE, Wang A, Lee DJ, et al; SOL Ojos Study Group. Visual impairment and cardiovascular risk factors in Hispanic and Latino adults. *JAMA Netw Open*. 2026;9(6):e2617975. doi:10.1001/jamanetworkopen.2026.17975

eMethods

eReferences

eFigure 1. Strengthening the Reporting of Observational Studies in Epidemiology (STROBE) Diagram: Hispanic Community Health Study/Study of Latinos (HCHC/SOL) and SOL Ojos Participant Flow

eFigure 2. Age-Standardized Prevalence of Major Cardiovascular Disease Risk Factors in SOL Ojos Study Population, by Hispanic/Latino Background

eFigure 3. Odds Ratios Estimates Between Major Cardiovascular Disease Risk Factors and Uncorrected Refractive Error in the SOL Ojos Study Population

eTable 1. Tabular Data Used to Generate Figure 1 and eFigure 2: Age-Standardized Prevalence and 95% CIs of Cardiovascular Disease Risk Factors of the SOL Ojos Study Population, by Hispanic/Latino Background

eTable 2. Tabular Data used to Generate Figure 2 and eFigure 3: Odds Ratio Estimates and 95% CIs of Visual Impairment in SOL Ojos Study Population

eTable 3. Final Model Estimates (Model 3) Used to Generate Figure 2 and eFigure 3: Odds Ratio Estimates and 95% CIs between Individual and Cumulative Cardiovascular Disease Risk Factors and Visual Impairment in SOL Ojos Population

This supplementary material has been provided by the authors to give readers additional information about their work.

© 2026 Joslin CE et al. *JAMA Network Open*.

eMethods:

Study Sample

Visit 1 (V1; 2008-2011) included a 7-hour baseline clinical exam;[1] Visit 2 (2014-2017) assessed risk factors for incident disease; and Visit 3 (V3; 2020-2024) evaluated associations between baseline health and subsequent fatal and nonfatal CVD and pulmonary events. Annual follow-up calls captured inter-visit hospitalizations and major clinical events.[2] HCHS/SOL has expanded to include 22 ancillary studies (2023),[2] providing disaggregated results that highlight heterogeneity within broader Hispanic/Latino groups.[3,4]

Examination Data

Visual acuity was scored as the total number of letters read correctly and converted to a logarithm of the minimum angle of resolution (logMAR), with "off-chart" visual acuity assigned logMAR values of 1.85 (count fingers) and 2.3 (hand motion).[5]

All data were recorded in the Research Electronic Data Capture (REDCap) system, hosted at the University of Illinois Chicago. REDCap is a secure, web-based software platform designed to support data capture for research studies, providing validated data entry, audit trails, and automated export procedures.[6,7]

Study Covariates

Participants self-reported sociodemographic factors (Hispanic/Latino background, age, sex, education, income, marital status, health insurance status), acculturation factors (nativity, years in the US, generational status, language preference), and medical history, including healthcare access in the prior 12 months. Current medications, including medications for diabetes, hypertension, and hyperlipidemia, were self-reported and recorded. Dietary intake was assessed by two 24-hour dietary recalls (6 weeks apart), with diet scores based on sex-specific quintiles of saturated fatty acids, potassium, calcium, and fiber intake and summed, with the highest 40th percentile indicating a healthier diet.[8,9] Data were collected at HCHS/SOL V3, except dietary intake (V1).

Age-Standardization Methods

We describe here the approach used to externally standardize the HCHS/SOL prevalences to the age distribution of the 2010 US Census population for SOL Ojos analyses. Because the standard 2010 US population includes individuals younger than 40 years, whereas the SOL Ojos study population was restricted to adults aged 40 years and older, we modified the census-based age distribution to align with the study eligibility criteria. Specifically, we derived age-specific weights using published 2010 US Census population estimates, restricted to individuals aged ≥40 years. Age-specific population distributions were obtained from the 2010 US Census (eTable 1)[1] and used to define the age strata for standardization.

To facilitate use of the 2010 US Census data for age adjustment, we categorized SOL Ojos population into four age groups, including: 40-49, 50-59, 60-69, and 70+, respectively.

Age-standardized prevalence estimates were calculated using the direct standardization method. Specifically, age-adjusted rates were computed as:

$$\sum_{i=1}^{n} r_i\left(\frac{p_i}{P}\right)$$

Where:

r_i = Rate in age group i in the population of interest
p_i = Standard population in age group i
$P = \sum_{i=1}^{n} p_i$
n = Total number of age groups over the age range of the age-adjusted rate.

© 2026 Joslin CE et al. *JAMA Network Open*.

Age-specific population counts and corresponding standardization weights were derived from the 2010 US Census, which follows.[10]

Population by Age and Sex: 2000 and 2010

(For information on confidentiality protection, nonsampling error, and definitions, see *www.census.gov/prod/cen2010/doc/sf1.pdf*)

Age	2000			2010			Percent change, 2000 to 2010		
	Both sexes	Male	Female	Both sexes	Male	Female	Both sexes	Male	Female
**All ages**	**281,421,906**	**138,053,563**	**143,368,343**	**308,745,538**	**151,781,326**	**156,964,212**	**9.7**	**9.9**	**9.5**
Under 5 years	19,175,798	9,810,733	9,365,065	20,201,362	10,319,427	9,881,935	5.3	5.2	5.5
5 to 9 years	20,549,505	10,523,277	10,026,228	20,348,657	10,389,638	9,959,019	–1.0	–1.3	–0.7
10 to 14 years	20,528,072	10,520,197	10,007,875	20,677,194	10,579,862	10,097,332	0.7	0.6	0.9
15 to 19 years	20,219,890	10,391,004	9,828,886	22,040,343	11,303,666	10,736,677	9.0	8.8	9.2
20 to 24 years	18,964,001	9,687,814	9,276,187	21,585,999	11,014,176	10,571,823	13.8	13.7	14.0
25 to 29 years	19,381,336	9,798,760	9,582,576	21,101,849	10,635,591	10,466,258	8.9	8.5	9.2
30 to 34 years	20,510,388	10,321,769	10,188,619	19,962,099	9,996,500	9,965,599	–2.7	–3.2	–2.2
35 to 39 years	22,706,664	11,318,696	11,387,968	20,179,642	10,042,022	10,137,620	–11.1	–11.3	–11.0
40 to 44 years	22,441,863	11,129,102	11,312,761	20,890,964	10,393,977	10,496,987	–6.9	–6.6	–7.2
45 to 49 years	20,092,404	9,889,506	10,202,898	22,708,591	11,209,085	11,499,506	13.0	13.3	12.7
50 to 54 years	17,585,548	8,607,724	8,977,824	22,298,125	10,933,274	11,364,851	26.8	27.0	26.6
55 to 59 years	13,469,237	6,508,729	6,960,508	19,664,805	9,523,648	10,141,157	46.0	46.3	45.7
60 to 64 years	10,805,447	5,136,627	5,668,820	16,817,924	8,077,500	8,740,424	55.6	57.3	54.2
65 to 69 years	9,533,545	4,400,362	5,133,183	12,435,263	5,852,547	6,582,716	30.4	33.0	28.2
70 to 74 years	8,857,441	3,902,912	4,954,529	9,278,166	4,243,972	5,034,194	4.7	8.7	1.6
75 to 79 years	7,415,813	3,044,456	4,371,357	7,317,795	3,182,388	4,135,407	–1.3	4.5	–5.4
80 to 84 years	4,945,367	1,834,897	3,110,470	5,743,327	2,294,374	3,448,953	16.1	25.0	10.9
85 to 89 years	2,789,818	876,501	1,913,317	3,620,459	1,273,867	2,346,592	29.8	45.3	22.6
90 to 94 years	1,112,531	282,325	830,206	1,448,366	424,387	1,023,979	30.2	50.3	23.3
95 to 99 years	286,784	58,115	228,669	371,244	82,263	288,981	29.5	41.6	26.4
100 years and over	50,454	10,057	40,397	53,364	9,162	44,202	5.8	–8.9	9.4
Median age	35.3	34.0	36.5	37.2	35.8	38.5	(X)	(X)	(X)

(X) Not applicable

Sources: U.S. Census Bureau, *Census 2000 Summary File 1* and *2010 Census Summary File 1*.

The extracted age groups and weights from the US Census data (above) used for SOL Ojos standardization are presented below.

Age groups and age-adjustment weights based on 2010 US Census Summary (above)

Age Group	Population	Adjustment Weights
40–49	43,599,555	0.3056435
50–59	41,962,930	0.2941704
60–69	29,253,187	0.2050720
70+	27,832,721	0.1951142

© 2026 Joslin CE et al. *JAMA Network Open*.

The SAS macro used to implement age standardization via PROC SURVEYREG is provided below.

```
%macro agestd_prev (data,bkg,age,var);
proc surveyreg data = &data order=internal;
strata strat;
cluster psu_id;
weight WEIGHT_NORM_OVERALL_OJOS;
domain dom;
class &bkg &age &var;
model &var = &bkg &age &bkg*&age / solution noint CLPARM;

estimate "Central American"
                        &bkg  1 0 0 0 0 0
                        &age 0.3056435 0.2941704 0.205072 0.1951142
                        &bkg*&age      0.3056435 0.2941704 0.205072 0.1951142
                                       0 0 0 0
                                       0 0 0 0
                                       0 0 0 0
                                       0 0 0 0
                                       0 0 0 0  / e;

estimate "Cuban"
                        &bkg  0 1 0 0 0 0
                        &age 0.3056435 0.2941704 0.205072 0.1951142
                        &bkg*&age      0 0 0 0
                                       0.3056435 0.2941704 0.205072 0.1951142
                                       0 0 0 0
                                       0 0 0 0
                                       0 0 0 0
                                       0 0 0 0  / e;

estimate "Mexican"
                        &bkg  0 0 1 0 0 0
                        &age 0.3056435 0.2941704 0.205072 0.1951142
                        &bkg*&age      0 0 0 0
                                       0 0 0 0
                                       0.3056435 0.2941704 0.205072 0.1951142
                                       0 0 0 0
                                       0 0 0 0
                                       0 0 0 0  / e;


estimate "Puerto-Rican"
                        &bkg  0 0 0 1 0 0
                        &age 0.3056435 0.2941704 0.205072 0.1951142
                        &bkg*&age      0 0 0 0
                                       0 0 0 0
                                       0.3056435 0.2941704 0.205072 0.1951142
                                       0 0 0 0
                                       0 0 0 0
                                       0 0 0 0  / e;

estimate "South American"
                        &bkg  0 0 0 0 1 0
                        &age 0.3056435 0.2941704 0.205072 0.1951142
```

© 2026 Joslin CE et al. *JAMA Network Open*.

```
                                &bkg*&age       0 0 0 0
                                                0 0 0 0
                                                0 0 0 0
                                                0 0 0 0
                                                0.3056435 0.2941704 0.205072 0.1951142
                                                        0 0 0 0  / e;

estimate "Other"
                                &bkg  0 0 0 0 0 1
                                &age 0.3056435 0.2941704 0.205072 0.1951142
                                &bkg*&age       0 0 0 0
                                                0 0 0 0
                                                0 0 0 0
                                                0 0 0 0
                                                0 0 0 0
                                        0.3056435 0.2941704 0.205072 0.1951142 / e;
run;
%mend agestd_prev;
```

© 2026 Joslin CE et al. *JAMA Network Open*.

<u>**eReferences**</u>

1. Sorlie PD, Aviles-Santa LM, Wassertheil-Smoller S, Kaplan RC, Daviglus ML, Giachello AL, Schneiderman N, Raij L, Talavera G, Allison M, Lavange L, Chambless LE, Heiss G. Design and implementation of the Hispanic Community Health Study/Study of Latinos. *Ann Epidemiol*. Aug 2010;20(8):629-41. doi:10.1016/j.annepidem.2010.03.015
2. Pirzada A, Cai J, Heiss G, Sotres-Alvarez D, Gallo LC, Youngblood ME, Aviles-Santa ML, Gonzalez HM, Isasi CR, Kaplan R, Kunz J, Lash JP, Lee DJ, Llabre MM, Penedo FJ, Rodriguez CJ, Schneiderman N, Sofer T, Talavera GA, Thyagarajan B, Wassertheil-Smoller S, Daviglus ML. Evolving Science on Cardiovascular Disease Among Hispanic/Latino Adults: JACC International. *J Am Coll Cardiol*. Apr 18 2023;81(15):1505-1520. doi:10.1016/j.jacc.2023.02.023
3. Ponce NA, Becker T, Shimkhada R. Breaking Barriers with Data Equity: The Essential Role of Data Disaggregation in Achieving Health Equity. *Annual review of public health*. Apr 2025;46(1):21-42. doi:10.1146/annurev-publhealth-072523-093838
4. Kauh TJ, Read JG, Scheitler AJ. The Critical Role of Racial/Ethnic Data Disaggregation for Health Equity. *Popul Res Policy Rev*. 2021;40(1):1-7. doi:10.1007/s11113-020-09631-6
5. Schulze-Bonsel K, Feltgen N, Burau H, Hansen L, Bach M. Visual Acuities “Hand Motion” and “Counting Fingers” Can Be Quantified with the Freiburg Visual Acuity Test. *Investigative Ophthalmology & Visual Science*. 2006;47(3):1236-1240. doi:10.1167/iovs.05-0981
6. Harris PA, Taylor R, Thielke R, Payne J, Gonzalez N, Conde JG. Research electronic data capture (REDCap)--a metadata-driven methodology and workflow process for providing translational research informatics support. *J Biomed Inform*. Apr 2009;42(2):377-81. doi:10.1016/j.jbi.2008.08.010
7. Harris PA, Taylor R, Minor BL, Elliott V, Fernandez M, O'Neal L, McLeod L, Delacqua G, Delacqua F, Kirby J, Duda SN, Consortium RE. The REDCap consortium: Building an international community of software platform partners. *J Biomed Inform*. Jul 2019;95:103208. doi:10.1016/j.jbi.2019.103208
8. Daviglus ML, Talavera GA, Aviles-Santa ML, Allison M, Cai J, Criqui MH, Gellman M, Giachello AL, Gouskova N, Kaplan RC, LaVange L, Penedo F, Perreira K, Pirzada A, Schneiderman N, Wassertheil-Smoller S, Sorlie PD, Stamler J. Prevalence of major cardiovascular risk factors and cardiovascular diseases among Hispanic/Latino individuals of diverse backgrounds in the United States. Multicenter Study
Research Support, N.I.H., Extramural. *JAMA*. Nov 7 2012;308(17):1775-84. doi:10.1001/jama.2012.14517
9. Liu K, Daviglus ML, Loria CM, Colangelo LA, Spring B, Moller AC, Lloyd-Jones DM. Healthy lifestyle through young adulthood and the presence of low cardiovascular disease risk profile in middle age: the Coronary Artery Risk Development in (Young) Adults (CARDIA) study. *Circulation*. Feb 28 2012;125(8):996-1004. doi:10.1161/CIRCULATIONAHA.111.060681
10. Howden LM, Meyer JA. Age and Sex Composition: 2010 -- Census.gov. U.S. CENSUS BUREAU; U.S. Department of Commerce, Economics and Statistics Administration. Accessed April 12, 2026. https://www.census.gov/library/publications/2011/dec/c2010br-03.html

© 2026 Joslin CE et al. *JAMA Network Open*.

<u>eFIGURE 1, Supplement.</u> STrengthening the Reporting of OBservational studies in Epidemiology (STROBE) Diagram for participant recruitment and flow in the Hispanic Community Health Study/Study of Latinos (HCHC/SOL) and SOL Ojos.

HCHS/SOL Visit 1 (V1; 2008-2011); Visit 2 (V2; 2014-2017); Visit 3 (V3; 2020-2024). Chicago = C, Miami = M.

© 2026 Joslin CE et al. *JAMA Network Open*.

STrengthening the Reporting of OBservational studies in Epidemiology (STROBE) Diagram

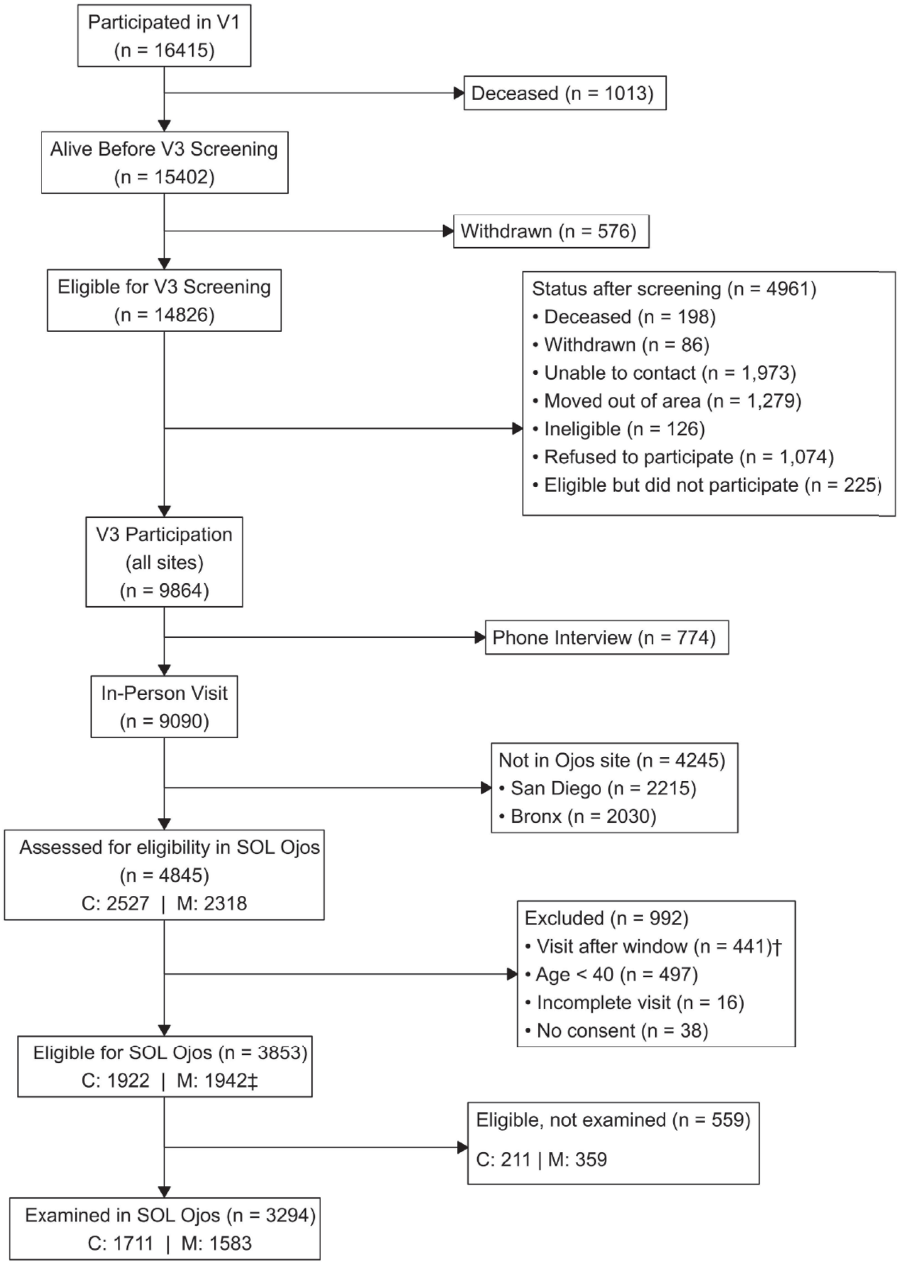

Counts are data-derived unless noted.

† Eligibility window uses V3 blood draw date ≤ 2023-06-17.

‡ Values verified from site logs where reasons could not be fully reconstructed from the analysis extract.

© 2026 Joslin CE et al. *JAMA Network Open*.

eFIGURE 2, Supplement. Bar Graphs Showing Age-Standardized Prevalence of Cardiovascular Disease Risk Factors at Visit 3 in the SOL Ojos Study Population, by Hispanic/Latino Background.

Estimates were weighted for study design and non-response and age-standardized to 2010 US Census population. eTable 1 (Supplement) presents tabular data used to generate the graphs. Whiskers indicate 95% CIs.

© 2026 Joslin CE et al. *JAMA Network Open*.

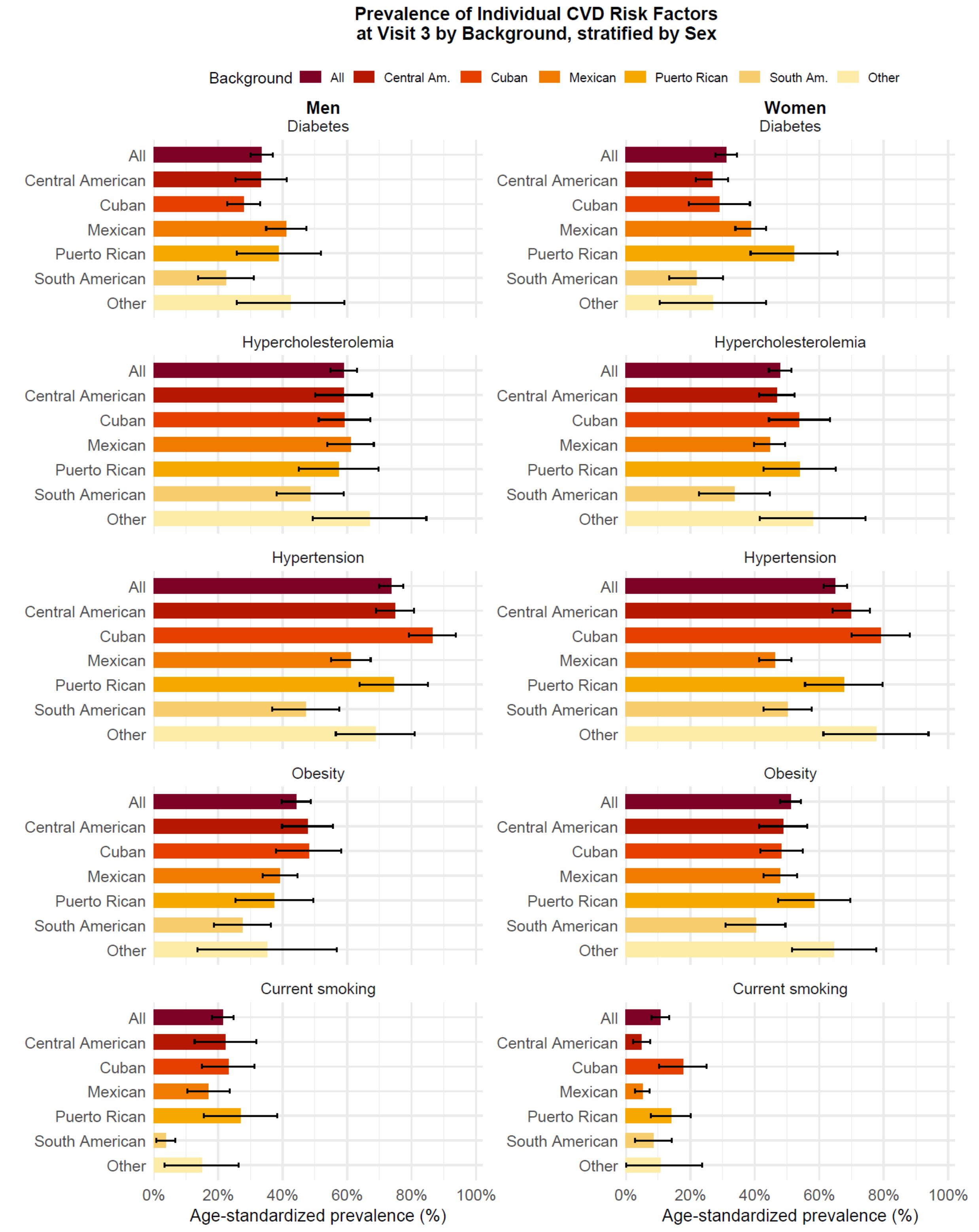

© 2026 Joslin CE et al. *JAMA Network Open*.

eFIGURE 3, Supplement. Forest Plots of Odds Ratio (OR) Estimates for Uncorrected Refractive Error Associated with Major Cardiovascular Disease (CVD) Risk Factors (RF) in SOL Ojos Study Population.

The ORs and 95% CIs were from nested, sequential modeling. eTable 2 (Supplement) presents the tabular data used to generate the forest plots. Larger data markers and tighter 95% CIs indicate greater precision. Model 1 adjusted for age, sex, Hispanic/Latino background, educational level, income, and marital status (traditional confounding). Model 2 adjusted for factors in model 1 plus all other CVD risk factors (biologic confounding – individual CVD risk factor exposures only due to collinearity with cumulative CVD risk factors). Model 3 adjusted for factors in model 2 plus diet, healthcare access, and nativity (contextual confounding related to nonmedical drivers of health relevant to Hispanic/Latino adults – thus, adjusted for all risk factor categories).

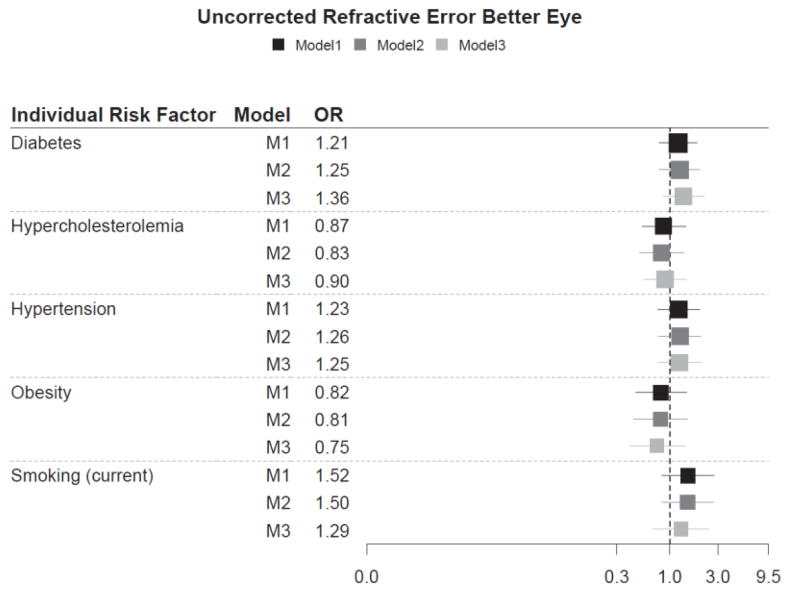

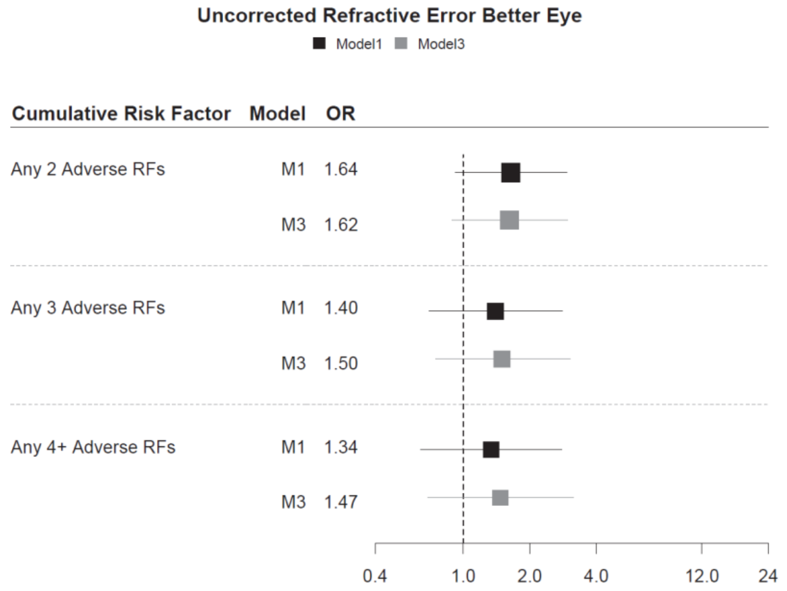

© 2026 Joslin CE et al. *JAMA Network Open*.

eTables:

eTable 1, Supplement. Tabular Data used to Generate Figure 1 and eFigure 2: Age-Standardized Prevalence of Cardiovascular Disease (CVD) Risk Factors in the SOL Ojos Study Population, by Hispanic/Latino Background[a]

Characteristics	Proportion of participants with CVD, weighted %(95%CI)							P-value[e]
	All	Central American	Cuban	Mexican	Puerto Rican	South American	Other[d]	
**Men, No.[b]**	1254	190	364	392	124	140	44	
**Individual CVD Risk Factors[c]**								
Diabetes	33.4 (30.0, 36.9)	33.3 (25.4, 41.3)	27.9 (22.8, 33.1)	41.1 (34.9, 47.4)	38.8 (25.7, 51.9)	22.4 (13.7, 31.1)	42.4 (25.8, 59.1)	0.002
Hypercholesterolemia	59.0 (54.9, 63.1)	58.9 (50.2, 67.7)	59.2 (51.2, 67.2)	61.1 (53.9, 68.3)	57.3 (45.0, 69.7)	48.6 (38.2, 59.0)	67.0 (49.3, 84.6)	0.21
Hypertension	73.7 (69.9, 77.5)	74.8 (68.9, 80.7)	86.5 (79.2, 93.8)	61.2 (55.1, 67.3)	74.4 (63.8, 85.1)	47.2 (36.7, 57.6)	68.7 (56.5, 81.0)	<0.001
Obesity	44.3 (39.7, 48.8)	47.7 (39.8, 55.6)	48.1 (38.0, 58.2)	39.2 (33.8, 44.6)	37.4 (25.3, 49.6)	27.5 (18.7, 36.3)	35.2 (13.6, 56.8)	0.006
Smoking (current)	21.4 (18.0, 24.8)	22.2 (12.7, 31.8)	23.2 (15.0, 31.3)	17.0 (10.4, 23.6)	27.0 (15.6, 38.3)	3.8 (0.8, 6.7)	14.9 (3.4, 26.4)	<0.001
**Cumulative CVD Risk Factors[c]**								
0 or any 1 Adverse RF	26.0 (22.0, 29.9)	22.7 (14.9, 30.4)	18.1 (10.5, 25.8)	28.3 (22.1, 34.5)	31.8 (21.5, 42.1)	52.2 (42.8, 61.5)	30.8 (18.6, 43.1)	<0.001
Any 2 Adverse RF	29.4 (25.7, 33.0)	30.9 (23.3, 38.6)	34.3 (26.0, 42.6)	28.6 (22.7, 34.4)	21.3 (12.8, 29.8)	28.5 (19.6, 37.4)	21.8 (4.3, 39.4)	0.45
Any 3 Adverse RF	27.9 (24.5, 31.4)	29.7 (20.9, 38.5)	31.0 (23.5, 38.6)	30.4 (24.3, 36.5)	32.0 (19.7, 44.4)	14.1 (8.0, 20.3)	24.9 (17.0, 32.8)	0.003
Any 4+ Adverse RF	16.7 (13.7, 19.8)	16.7 (10.4, 23.0)	16.5 (12.1, 20.9)	12.8 (8.9, 16.6)	14.8 (4.0, 25.6)	5.2 (2.0, 8.5)	22.4 (6.0, 38.8)	<0.001
**Women, No.[b]**	2034	399	487	621	200	260	67	
**Individual CVD Risk Factors[c]**								
Diabetes	31.1 (27.8, 34.4)	26.8 (21.8, 31.8)	29.0 (19.6, 38.5)	38.8 (34.0, 43.6)	52.2 (38.7, 65.7)	21.9 (13.5, 30.2)	27.0 (10.5, 43.5)	<0.001
Hypercholesterolemia	47.9 (44.4, 51.4)	46.8 (41.4, 52.3)	53.8 (44.4, 63.3)	44.6 (39.7, 49.4)	53.9 (42.8, 65.1)	33.7 (22.7, 44.7)	58.0 (41.6, 74.3)	0.01
Hypertension	65.0 (61.4, 68.6)	69.9 (64.1, 75.8)	79.1 (70.0, 88.1)	46.3 (41.3, 51.4)	67.6 (55.6, 79.7)	50.2 (42.8, 57.7)	77.6 (61.3, 93.9)	<0.001
Obesity	51.2 (47.9, 54.4)	48.8 (41.3, 56.3)	48.3 (41.7, 54.9)	47.9 (42.7, 53.1)	58.4 (47.3, 69.6)	40.3 (31.0, 49.5)	64.6 (51.5, 77.7)	0.022
Smoking (current)	10.7 (7.9, 13.5)	4.9 (2.3, 7.6)	17.7 (10.4, 25.0)	5.2 (2.9, 7.4)	14.0 (7.8, 20.2)	8.6 (2.9, 14.3)	10.8 (0.0, 23.7)	0.002
**Cumulative CVD Risk Factors[c]**								
0 or any 1 Adverse RF	33.8 (30.3, 37.3)	36.6 (31.7, 41.5)	25.0 (15.9, 34.1)	44.5 (38.6, 50.4)	19.3 (8.0, 30.6)	51.7 (40.1, 63.3)	23.8 (7.7, 39.8)	<0.001
Any 2 Adverse RF	29.5 (26.5, 32.6)	32.9 (27.0, 38.9)	31.1 (23.4, 38.8)	24.4 (18.8, 30.1)	29.2 (18.9, 39.5)	26.5 (18.5, 34.4)	24.6 (14.8, 34.4)	0.27
Any 3 Adverse RF	24.2 (20.9, 27.5)	20.5 (15.3, 25.7)	28.5 (19.5, 37.5)	20.0 (16.1, 23.9)	32.6 (21.0, 44.2)	16.0 (7.1, 24.8)	38.2 (24.2, 52.3)	0.047
Any 4+ Adverse RF	12.5 (10.4, 14.5)	10.0 (6.4, 13.6)	15.4 (9.6, 21.2)	11.1 (8.3, 13.8)	18.9 (11.4, 26.4)	5.9 (2.6, 9.2)	13.4 (0.9, 25.9)	0.02

[a] Values (except for N) were weighted for study design and non-response, and age-standardized to Census 2010 US population.
[b] N represents the number of participants with non-missing values background. Missingness of background, n=6 (excluded from analysis, 6/3294 = 0.2%).
[c] Risk factors were defined as follows: hypercholesterolemia: total cholesterol >240 mg/dL, HDL <40 mg/dL, LDL >160 mg/dL, or on treatment; hypertension: systolic/diastolic blood pressure >130/>80 or on treatment; current smoking: currently smoking and having smoked >100 cigarettes over lifetime; diabetes mellitus: use of diabetes medications, fasting glucose >126 mg/dL or ≥200 mg/dL for ≤8 hours, or HbA1c >6.5%; obesity: BMI >30kg/m2. Participants were classified as having 0, any 1 only, any 2, any 3, or any 4+ adverse CVD RF as defined above.
[d] Dominican participants were grouped as “Other” to improve stability, which also included those reporting multiple backgrounds.
[e] P-value based on Wald tests across all background groups.

© 2026 Joslin CE et al. *JAMA Network Open*.

eTable 2, Supplement. Tabular Data used to Generate Figure 2 and eFigure 3: Odds Ratio Estimates and 95% Confidence Interval (95%CI) of Visual Impairment[a] in the SOL Ojos Study Population, Stratified by Individual and Cumulative Cardiovascular Disease (CVD) Risk Factors (RF). Models increasingly adjust for Various Factors that are Non-Medical Drivers of Health (NMDOH).

	Visual Impairment								
	Habitual Visual Acuity[a,b]			Best-Corrected Visual Acuity[a,b]			URE Better Eye[a]		
**Characteristics**	**Model 1[d]**	**Model 2[e]**	**Model 3[f]**	**Model 1[d]**	**Model 2[e]**	**Model 3[f]**	**Model 1[d]**	**Model 2[e]**	**Model 3[f]**
Individual CVD Risk Factors[c]									
Diabetes	2.0 (1.4, 2.8)	1.9 (1.3, 2.8)	2.0 (1.4, 3.0)	6.1 (3.2, 11.4)	4.9 (2.6, 9.4)	4.6 (2.4, 8.9)	1.2 (0.8, 1.9)		1.4 (0.8, 2.2)
Hypercholesterolemia	1.1 (0.7, 1.7)	1.0 (0.6, 1.5)	1.0 (0.7, 1.6)	1.9 (1.0, 3.8)	1.5 (0.7, 2.9)	1.5 (0.8, 2.9)	0.9 (0.5, 1.5)	0.8 (0.5, 1.4)	0.9 (0.5, 1.5)
Hypertension	1.3 (0.8, 2.1)	1.2 (0.7, 1.9)	1.2 (0.7, 1.9)	1.7 (0.5, 5.3)	1.2 (0.4, 3.5)	1.2 (0.4, 3.5)	1.2 (0.8, 2.0)	1.3 (0.8, 2.0)	1.3 (0.8, 2.1)
Obesity	1.1 (0.7, 1.8)	1.0 (0.6, 1.6)	1.0 (0.6, 1.6)	2.2 (1.0, 4.7)	1.6 (0.7, 3.6)	1.7 (0.8, 3.7)	0.8 (0.5, 1.5)	0.8 (0.4, 1.5)	0.8 (0.4, 1.4)
Smoking (current)	1.1 (0.6, 1.9)	1.1 (0.6, 1.9)	0.9 (0.5, 1.7)	0.3 (0.1, 1.4)	0.3 (0.0, 1.6)	0.2 (0.0, 1.8)	1.5 (0.8, 2.7)	1.5 (0.8, 2.7)	1.3 (0.7, 2.5)
Cumulative CVD Risk Factors[c]									
1 or less Adverse RF (ref)									
Any 2 Adverse RF	1.7 (1.0, 2.9)		1.7 (1.0, 3.0)	2.3 (0.9, 6.0)		2.2 (0.9, 5.5)	1.6 (0.9, 2.9)		1.6 (0.9, 3.0)
Any 3 Adverse RF	1.8 (1.0, 3.1)		1.8 (1.0, 3.3)	3.6 (1.5, 8.8)		3.5 (1.4, 8.3)	1.4 (0.7, 2.8)		1.5 (0.7, 3.0)
Any 4+ Adverse RF	2.7 (1.5, 4.7)		2.8 (1.5, 5.2)	10.5 (4.7, 23.5)		9.8 (4.3, 22.1)	1.3 (0.6, 2.8)		1.5 (0.7, 3.2)

[a] Defined as visual acuity less than or equal to 20/40 in the better-seeing eye; Uncorrected Refractive Error (URE) defined as habitual visual acuity of worse than or equal to 20/40 that improves to better than 20/40 best-corrected visual acuity in the better-seeing eye.

[b] VI as measured by best-corrected visual acuity determined following ETDRS refraction; VI as measured by habitual visual acuity determined according to presenting correction.

[c] Risk factors were defined as follows: hypercholesterolemia: total cholesterol >240 mg/dL, HDL <40 mg/dL, LDL >160 mg/dL, or on treatment; hypertension: systolic/diastolic blood pressure >130/>80 or on treatment; current smoking: currently smoking and having smoked >100 cigarettes over lifetime; diabetes mellitus: use of diabetes medications, fasting glucose >126 mg/dL or ≥200 mg/dL for ≤8 hours, or HbA1c >6.5%; obesity: BMI >30kg/m2. Participants were classified as having 0-1, any 2, any 3, or any 4+ adverse risk factors as defined above.

[d] Model 1 adjusted for age, sex, Hispanic/Latino background, educational level, income, and marital status. Income was recategorized as a 3-cateory variable: <$20,000, $20,001-$50,000, and >$50,000.

[e] Model 2 adjusted for factors in model 1 plus all other CVD risk factors

[f] Model 3 adjusted for factors in model 2 plus diet, healthcare access, and nativity.

© 2026 Joslin CE et al. *JAMA Network Open*.

eTable 3 Supplement. Final Model Estimates (Model 3) used to Generate Figure 2 and eFigure 3: Odds Ratio Estimates and 95% Confidence Interval (95%CI) between Individual and Cumulative Cardiovascular Disease (CVD) Risk Factors (RF) and Visual Impairment[a] in the SOL Ojos Population

	Visual Impairment					
	Habitual Acuity[a,b]		Best-Corrected Acuity[a,b]		URE Better Eye[a]	
**Characteristics**	**Individual RF**	**Cumulative RF**	**Individual RF**	**Cumulative RF**	**Individual RF**	**Cumulative RF**
Individual CVD Risk Factors	1		1		1	
Diabetes	2.0 (1.4, 3.0)		4.5 (2.4, 8.6)		1.4 (0.8, 2.2)	
Hypertension	1.2 (0.7, 1.9)		1.2 (0.4, 3.3)		1.3 (0.8, 2.1)	
Hypercholesterolemia	1.0 (0.7, 1.5)		1.5 (0.8, 2.9)		0.9 (0.5, 1.4)	
Obesity						
Underweight + Normal (ref)	1		1		1	
Overweight	0.8 (0.4, 1.3)		0.8 (0.3, 1.8)		0.8 (0.4, 1.5)	
Obese	0.9 (0.6, 1.6)		1.8 (0.8, 4.0)		0.7 (0.4, 1.4)	
Current Smoker	1.0 (0.5, 1.8)		0.2 (0.0, 1.8)		1.4 (0.7, 2.5)	
Cumulative CVD Risk Factors						
1 or less Adverse RF (ref)		1		1		1
Any 2 Adverse RF		1.7 (1.0, 3.0)		2.1 (0.8, 5.4)		1.7 (0.9, 3.0)
Any 3 Adverse RF		1.8 (1.0, 3.3)		3.4 (1.4, 8.3)		1.5 (0.7, 3.0)
Any 4+ Adverse RF		2.7 (1.5, 5.1)		9.3 (4.0, 21.3)		1.5 (0.7, 3.1)
Background						
South American (ref)	1	1	1	1	1	1
Central American	0.8 (0.4, 1.7)	0.7 (0.3, 1.7)	0.3 (0.1, 1.1)	0.3 (0.1, 1.1)	1.2 (0.5, 2.9)	1.2 (0.5, 2.9)
Mexican	1.8 (1.0, 3.4)	1.9 (1.0, 3.6)	0.6 (0.2, 1.5)	0.6 (0.3, 1.5)	3.1 (1.5, 6.3)	3.1 (1.5, 6.3)
Cuban	0.5 (0.2, 1.0)	0.4 (0.2, 0.9)	0.3 (0.1, 0.8)	0.3 (0.1, 0.7)	0.6 (0.3, 1.4)	0.6 (0.3, 1.4)
Puerto Rican	0.7 (0.3, 1.5)	0.7 (0.4, 1.6)	0.6 (0.2, 1.6)	0.6 (0.2, 1.5)	0.8 (0.3, 1.8)	0.8 (0.4, 1.9)
Other	1.4 (0.4, 4.8)	1.4 (0.4, 4.8)	0.0 (0.0, 0.0)	0.0 (0.0, 0.0)	3.3 (1.0, 11.0)	3.2 (1.0, 10.2)
Sex						
Men (ref)	1	1	1	1	1	1
Women	0.8 (0.6, 1.2)	0.8 (0.6, 1.2)	0.8 (0.4, 1.5)	1.0 (0.5, 1.8)	0.8 (0.5, 1.2)	0.7 (0.4, 1.2)
Age (1 year)	1.0 (1.0, 1.1)	1.1 (1.0, 1.1)	1.1 (1.0, 1.2)	1.1 (1.0, 1.2)	1.0 (1.0, 1.1)	1.0 (1.0, 1.1)
Marital Status						
Single (ref)	1	1	1	1	1	1
Married/Living with a Partner	0.6 (0.4, 1.1)	0.6 (0.4, 1.2)	0.5 (0.2, 1.2)	0.6 (0.2, 1.4)	0.7 (0.4, 1.4)	0.7 (0.4, 1.3)
Separated/Divorced/Widow	0.6 (0.3, 1.0)	0.6 (0.3, 1.0)	0.5 (0.2, 1.1)	0.6 (0.3, 1.5)	0.6 (0.3, 1.1)	0.6 (0.3, 1.1)
Education						
Greater than High School (ref)	1	1	1	1	1	1
Less than high school	1.4 (0.9, 2.2)	1.4 (0.9, 2.2)	2.9 (1.1, 7.5)	2.9 (1.2, 7.2)	0.9 (0.6, 1.5)	0.9 (0.6, 1.5)
High school graduate	1.2 (0.7, 1.8)	1.2 (0.7, 1.9)	1.1 (0.4, 3.0)	1.0 (0.3, 2.8)	1.1 (0.6, 2.0)	1.2 (0.6, 2.0)
Income						
$50,000+ (ref)	1	1	1	1	1	1
$20,001-$25,000	2.2 (0.9, 5.5)	2.4 (1.0, 5.8)	2.2 (0.5, 9.9)	2.3 (0.5, 10.3)	2.3 (0.9, 6.3)	2.5 (0.9, 6.9)
$25,001-$50,000	1.8 (0.7, 4.4)	1.9 (0.8, 4.5)	1.9 (0.5, 6.9)	2.2 (0.6, 7.7)	1.7 (0.6, 4.8)	1.8 (0.6, 5.0)
Nativity						
US Born (ref)	1	1	1	1	1	1
Less than 20 years	1.5 (0.5, 4.1)	1.4 (0.5, 3.9)	3.3 (0.3, 33.6)	3.1 (0.3, 30.4)	1.4 (0.5, 4.2)	1.3 (0.4, 3.9)
20 Years or more	1.8 (0.7, 4.7)	1.7 (0.7, 4.6)	3.9 (0.4, 35.3)	3.8 (0.4, 32.0)	1.7 (0.6, 5.0)	1.6 (0.5, 4.6)
Diet Score						
>= 60th percentile (ref)	1	1	1	1	1	1
< 60th percentile	1.3 (0.8, 2.0)	1.2 (0.8, 1.9)	1.0 (0.5, 1.8)	0.9 (0.5, 1.7)	1.4 (0.8, 2.4)	1.4 (0.8, 2.4)

[a] Defined as visual acuity less than or equal to 20/40 in the better-seeing eye; Uncorrected Refractive Error (URE) defined as habitual visual acuity of worse than or equal to 20/40 that improves to better than 20/40 best-corrected visual acuity in the better-seeing eye.
[b] VI as measured by best-corrected visual acuity as determined following ETDRS refraction; VI as measured by habitual visual acuity determined according to presenting correction.
[c] Risk factors were defined as follows: hypercholesterolemia: total cholesterol >240 mg/dL, HDL <40 mg/dL, LDL >160 mg/dL, or on treatment; hypertension: systolic/diastolic blood pressure >130/>80 or on treatment; current smoking: currently smoking and having smoked >100 cigarettes over lifetime; diabetes mellitus: use of diabetes medications, fasting glucose >126 mg/dL or ≥200 mg/dL for ≤8 hours, or HbA1c >6.5%; obesity: BMI >30kg/m2. Participants were classified as having 0, any 1 only, any 2, any 3, or any 4+ adverse RF as defined above.
[d] Model 1 adjusted for age, sex, Hispanic/Latino background, educational level, income, and marital status. Income was recategorized as a 3-cateory variable: <$20,000, $20,001-$50,000, and >$50,000 (not shown).
[e] Model 2 adjusted for factors in model 1 plus all other CVD risk factors (not shown).
[f] Model 3 adjusted for factors in model 2 plus diet, healthcare access, and nativity.

© 2026 Joslin CE et al. *JAMA Network Open*.
