# Supplementary material for: Visual Impairment and Cardiovascular Risk Factors in Hispanic and Latino Adults
Source: JAMA Netw Open. 2026 Jun 12;9(6):e2617975. doi: 10.1001/jamanetworkopen.2026.17975 (PMC13263780; doi:10.1001/jamanetworkopen.2026.17975)
Supplement: Supplement 2. — Members of the SOL Ojos Study Group [file jamanetwopen-e2617975-s002.pdf]

*Group Name(s): SOL Ojos Study Group							
*First Name and Middle Initial(s)	*Last Name	*Suffix (eg, Jr, III)	Academic Degrees	Institution	Location (city, state/province, country)	Role or Contribution, eg, chair, principal investigator	Group (if more than 1 Group listed in the byline) and/or Subgroup (eg, Steering Committee)
Charlotte E.	Joslin		OD, PhD	University of Illinois Chicago	Chicago, IL	Co-Principal Investigator	UIC SOL Ojos
Giselle A.	Gutierrez Savoy		MPH	University of Illinois Chicago	Chicago, IL	Project Director	UIC SOL Ojos
Heather	Pauls Hrynyk		MPH	University of Illinois Chicago	Chicago, IL	Project Director (2019-2020)	UIC SOL Ojos
Angie	Wang		PhD, MS	University of Illinois Chicago	Chicago, IL	Biostatistician	UIC SOL Ojos
Michael L.	Stewart		ScM	University of Illinois Chicago	Chicago, IL	Biostatistician	UIC SOL Ojos
Raiza	Perez Lucena		MD	University of Illinois Chicago	Chicago, IL	Research Examiner; Glaucoma Outcomes Reviewer	UIC SOL Ojos
Chunyu	Guo		MD, PhD, MPH	University of Illinois Chicago	Chicago, IL	Research Examiner (2020-2023)	UIC SOL Ojos
Evelyn	Ramirez		COT	University of Illinois Chicago	Chicago, IL	Ophthalmic Technician (2021-2023)	UIC SOL Ojos
Patrizia A.	Chavero		MD	University of Illinois Chicago	Chicago, IL	Recruiter/Interviewer (2022-2023)	UIC SOL Ojos
Lucila	Suarez		MSW	University of Illinois Chicago	Chicago, IL	Recruiter/Interviewer (2022-2023)	UIC SOL Ojos
Banelly	Mora		BS	University of Illinois Chicago	Chicago, IL	Recruiter/Interviewer (2022-2023)	UIC SOL Ojos
Sydney	Tobias		MS	University of Illinois Chicago	Chicago, IL	Biostatistician (2024)	UIC SOL Ojos
Lawrence J.	Ulanski		MD	University of Illinois Chicago	Chicago, IL	Co-Investigator	UIC SOL Ojos
Thasarat S.	Vajaranant		MD, MHA	University of Illinois Chicago	Chicago, IL	Co-Investigator; Glaucoma Outcomes Reviewer	UIC SOL Ojos
Manishi A.	Desai		MD	Boston University	Boston, MA	Glaucoma Outcomes Reviewer	UIC SOL Ojos
Pathik P.	Amin		OD	University of Illinois Chicago	Chicago, IL	Glaucoma Outcomes Reviewer	UIC SOL Ojos
Hannah	Yoon		OD, MS	University of Illinois Chicago	Chicago, IL	Glaucoma Outcomes Reviewer	UIC SOL Ojos
Charles W.	Kinnaird		OD	University of Illinois Chicago	Chicago, IL	Glaucoma Outcomes Reviewer	UIC SOL Ojos
Martha L.	Daviglus		MD, PhD	University of Illinois Chicago	Chicago, IL	HCHS/SOL Chicago Field Center Site PI	UIC SOL Ojos
Ramón A.	Durazo-Arvizu		PhD	University of Illinois Chicago	Chicago, IL	HCSH/SOL Chicago Field Center: Co-I, biostatistician (2019-2022)	UIC SOL Ojos
Margaret	Weiss		MD, PhD	University of Illinois Chicago	Chicago, IL	SOL Ojos Clinic Volunteer (2022-2023)	UIC SOL Ojos
Norma	Del Risco		MD	University of Illinois Chicago	Chicago, IL	SOL Ojos Clinic Volunteer (2022-2023)	UIC SOL Ojos
David J.	Lee		PhD	The University of Miami	Miami, FL	Co-Principal Investigator	UM SOL Ojos
Stephanie	Negron		MSPH	The University of Miami	Miami, FL	UM Site Project Coordinator	UM SOL Ojos
Byron	Lam		MD	The University of Miami	Miami, FL	Co-Investigator	UM SOL Ojos

Carlos E.	Mendoza-Santiesteban		MD, PhD	The University of Miami	Miami, FL	Co-Investigator	UM SOL Ojos
Diane	Zheng		PhD	The University of Miami	Miami, FL	Biostatistician/Epidemiologist (2020-2023)	UM SOL Ojos
Neil	Schneiderman		PhD	The University of Miami	Miami, FL	HCHS/SOL Miami Field Center Site PI (2020-2023)	UM SOL Ojos
Laura A.	McClure		MSPH	The University of Miami	Miami, FL	Miami Site Project Coordinator (2019-2022)	UM SOL Ojos
Miguel	Valladares Regalado		MD	The University of Miami	Miami, FL	Research Examiner (2020-2023)	UM SOL Ojos
Tamara	Juvier-Riesgo		MD, MS, PhD	The University of Miami	Miami, FL	Research Examiner (2021-2023)	UM SOL Ojos
Liliana	Rosello-Rodriguez		MD, MSN, APRN, FNP-BC	The University of Miami	Miami, FL	Research Examiner (2021-2023)	UM SOL Ojos
Estefania	Ruano-Herreria		MPH	The University of Miami	Miami, FL	Recruiter/Interviewer (2020-2022)	UM SOL Ojos
Maia	Junco		MPH, MA, PhD	The University of Miami	Miami, FL	Recruiter/Interviewer (2022-2023)	UM SOL Ojos
Diana	Hernandez Payano		MPH	The University of Miami	Miami, FL	Recruiter/Interviewer (2022-2023)	UM SOL Ojos
Richard	Ramos		MPH	The University of Miami	Miami, FL	Recruiter/Interviewer (2022-2023)	UM SOL Ojos
Eliseo	Moreno Perez		MD	The University of Miami	Miami, FL	Recruiter/Interviewer (2022-2023)	UM SOL Ojos
Maria Cristina	Buhl		MD	The University of Miami	Miami, FL	Recruiter/Interviewer (2020-2022)	UM SOL Ojos
Jianwen	Cai		PhD	University of North Carolina at Chapel Hill	Chapel Hill, NC	Biostatistican	HCHS/SOL Coordinating Center
Franklyn	Gonzales	II	MS	University of North Carolina at Chapel Hill	Chapel Hill, NC	Biostatistican	HCHS/SOL Coordinating Center
Marston	Youngblood Jr.		MA, MPH	University of North Carolina at Chapel Hill	Chapel Hill, NC	IRB Coordinator	HCHS/SOL Coordinating Center
Maryann	Redford		DDS, MPH	NIH/NEI	Bethesda, MD	Program Officer	National Eye Institute (NEI), National Institutes of Health (NIH)
Jimmy T.	Le		ScD	NIH/NEI	Bethesda, MD	Program Officer	National Eye Institute (NEI), National Institutes of Health (NIH)
Karla	Zadnik		OD, PhD	The Ohio State University	Columbus, OH	Data Monitoring Oversight Committee Chair	Data Monitoring Overight Committee
Juan E.	Grunwald		MD, PhD	University of Pennsylvania	Philadelphia, PA	Data Monitoring Oversight Committee Member	Data Monitoring Overight Committee
Henry D.	Jampel		MD, MHS	Johns Hopkins University	Baltimore, MD	Data Monitoring Oversight Committee Member	Data Monitoring Overight Committee
Aracely	Rosales			N/A	Miami Beach, FL	Data Monitoring Oversight Committee Member	Data Monitoring Overight Committee
Brisa N.	Sánchez		PhD	Drexel University	Philadelphia, PA	Data Monitoring Oversight Committee Member	Data Monitoring Overight Committee
Sheila K.	West		PhD PharmD	Johns Hopkins University	Baltimore, MD	Data Monitoring Oversight Committee Member	Data Monitoring Overight Committee
Siew Wei Gavin	Tan		MBBS, M Med(Ophth), MRCSEd, FRCS(Ed), FAMS, PhD	Singapore National Eye Centre	Singapore	Ocular Reading Centre	Singapore National Eye Centre
Yi Chong	Teo		MBBS, MMed (Ophth), FAMS, PhD	Singapore National Eye Centre	Singapore	Ocular Reading Centre	Singapore National Eye Centre
Haslina	Hamzah		BSc	Singapore National Eye Centre	Singapore	Ocular Reading Centre; First Reader(ONH); Project Manager	Singapore National Eye Centre

Shu Chin Serene	Sim		BSc	Singapore National Eye Centre	Singapore	Ocular Reading Centre; First Reader(FAF)	Singapore National Eye Centre
Amalia	Juhari		Dip	Singapore National Eye Centre	Singapore	Ocular Reading Centre; First Reader(OCT)	Singapore National Eye Centre
Muhamad Asri	Abdul Kadir		Dip	Singapore National Eye Centre	Singapore	Ocular Reading Centre; First Reader(DR); Project Coordinator	Singapore National Eye Centre
Raudhah Hanim	Mohamed Yusof		BA	Singapore National Eye Centre	Singapore	Ocular Reading Centre; First Reader(AMD)	Singapore National Eye Centre
Xingxiu	Ho		BSc	Singapore National Eye Centre	Singapore	Ocular Reading Centre; Second Reader(AMD)	Singapore National Eye Centre
Jinyi	Ho		Dip	Singapore National Eye Centre	Singapore	Ocular Reading Centre; Second Reader(OCT)	Singapore National Eye Centre
Nishal Banu	Makdoom		Bcou	Singapore National Eye Centre	Singapore	Ocular Reading Centre; Second Reader(DR)	Singapore National Eye Centre
Kayathri	Jaya Paul		Dip	Singapore National Eye Centre	Singapore	Ocular Reading Centre; QC Repro Reader(ONH)	Singapore National Eye Centre
Sharifah Athirah Diba	Sagoff		Dip	Singapore National Eye Centre	Singapore	Ocular Reading Centre; Second Reader(FAF)	Singapore National Eye Centre
