# Supplementary material for: Visual Impairment and Cardiovascular Risk Factors in Hispanic and Latino Adults
Source: JAMA Netw Open. 2026 Jun 12;9(6):e2617975. doi: 10.1001/jamanetworkopen.2026.17975 (PMC13263780; doi:10.1001/jamanetworkopen.2026.17975)
Supplement: Supplement 3. — Data Sharing Statement [file jamanetwopen-e2617975-s003.pdf]

Data Sharing Statement

Joslin. Visual Impairment and Cardiovascular Risk Factors in Hispanic and Latino Adults. *JAMA Netw Open*. Published June 12, 2026. doi:10.1001/jamanetworkopen.2026.17975

Data
Data available: Yes
Data types: Deidentified participant data, Data dictionary
How to access data: dbGaP under the Hispanic Community Health Study/Study of Latinos (HCHS/SOL)
When available: With publication

Supporting Documents
Document types: None

Additional Information
Who can access the data: Data will be available on dbGaP under the Hispanic Community Health Study/Study of Latinos (HCHS/SOL).
Types of analyses: Any purpose.
Mechanisms of data availability: Data will be made available through dbGaP as controlled-access data upon approval of a Data Access Request and execution of a Data Use Certification agreement, without direct investigator support.
